# Population genomic datasets describing the post-vaccine evolutionary epidemiology of *Streptococcus pneumoniae*

**DOI:** 10.1038/sdata.2015.58

**Published:** 2015-10-27

**Authors:** Nicholas J. Croucher, Jonathan A. Finkelstein, Stephen I. Pelton, Julian Parkhill, Stephen D. Bentley, Marc Lipsitch, William P. Hanage

**Affiliations:** 1 Department of Infectious Disease Epidemiology, Imperial College London, St Mary’s Campus, London W2 1pg, UK; 2 Department of Population Medicine, Harvard Medical School and Harvard Pilgrim Health Care Institute, Boston, Massachusetts 02215, USA; 3 Division of General Pediatrics, Boston Children's Hospital, Boston, Massachusetts 02215, USA; 4 Maxwell Finland Laboratory for Infectious Diseases, Boston University Medical Center, Boston, Massachusetts 02118, USA; 5 Pathogen Genomics, The Wellcome Trust Sanger Institute, Wellcome Trust Genome Campus, Cambridge CB10 1SA, UK; 6 Center for Communicable Disease Dynamics, Harvard T.H. Chan School of Public Health, 677 Huntington Avenue, Boston, Massachusetts 02115, USA

**Keywords:** Bacterial genetics, Molecular evolution, Genetic variation, Respiratory tract diseases

## Abstract

*Streptococcus pneumoniae* is common nasopharyngeal commensal bacterium and important human pathogen. Vaccines against a subset of pneumococcal antigenic diversity have reduced rates of disease, without changing the frequency of asymptomatic carriage, through altering the bacterial population structure. These changes can be studied in detail through using genome sequencing to characterise systematically-sampled collections of carried *S. pneumoniae*. This dataset consists of 616 annotated draft genomes of isolates collected from children during routine visits to primary care physicians in Massachusetts between 2001, shortly after the seven valent polysaccharide conjugate vaccine was introduced, and 2007. Also made available are a core genome alignment and phylogeny describing the overall population structure, clusters of orthologous protein sequences, software for inferring serotype from Illumina reads, and whole genome alignments for the analysis of closely-related sets of pneumococci. These data can be used to study both bacterial evolution and the epidemiology of a pathogen population under selection from vaccine-induced immunity.

## Background & Summary

*Streptococcus pneumoniae* (the pneumococcus) is a genetically diverse bacterial species commonly asymptomatically carried in the nasopharynx of infants, an age group not capable of generating a strong adaptive immune response to the polysaccharide capsule that envelopes most pneumococcal cells^1^. This capsule inhibits immune clearance by both complement- and neutrophil-mediated pathways^2^, and is a critical factor in allowing *S. pneumoniae* to invade other anatomical sites and cause disease such as pneumonia, bacteraemia and meningitis, particularly in both the young and elderly. Consequently, polysaccharide conjugate vaccines (PCVs) based on the pneumococcal capsule have been developed to induce anti-pneumococcal immunity in children^3^. These vaccine formulations are limited in the number of antigens they contain: the first licensed formulation (PCV7) contained seven serotypes. However, over 90 immunologically distinct capsule polysaccharides (serotypes) have been identified in *S. pneumoniae* ^4^, which is the consequence of extensive genetic variation at the capsule polysaccharide synthesis (*cps*) locus. Hence at the point of vaccine introduction the pneumococcal population consists of a mix of ‘vaccine serotypes’, susceptible to artificially-induced host immunity, and ‘non-vaccine serotypes’. Nevertheless, the widespread use of PCVs has caused a substantial fall in the incidence of invasive pneumococcal disease^5^.

This is the consequence of the PCV7 vaccine having at least 50% efficacy in preventing nasopharyngeal carriage of vaccine serotypes^6,7^, and around 98% (ref. 8) efficacy against invasive disease caused by the same types. However, the overall levels of pneumococcal carriage have not changed post-vaccination owing to serotype replacement^9,10^: the increase in frequency of non-vaccine serotypes. The reduced incidence of pneumococcal disease has therefore been attributed to the lower rate at which these non-vaccine serotypes cause symptomatic infections relative to vaccine serotypes^11^. Additionally, as many multidrug-resistant pneumococci were of PCV7 serotypes prior to the vaccine’s introduction, it was anticipated that PCV7 would decrease the levels of *S. pneumoniae* antimicrobial resistance; however, any such benefit in this regard observed shortly after vaccine introduction^12^ was not sustained over the longer term^13^. Hence understanding how the carried pneumococcal population structure changes following the implementation of partial coverage PCVs is important for relating the intervention to its subsequent clinical outcomes.

To address this question, the carried population of pneumococci in Massachusetts has been followed by the *Streptococcus pneumoniae* Antimicrobial Resistance in Children (SPARC) project. The instigation of this study coincided with the introduction of PCV7 in the USA in 2000. Samples were obtained through swabbing the nasopharynx of children seven years of age or under during routine visits to primary care physicians^14^. In the spring of 2001, winter and spring of 2004, and winter and spring of 2006–2007, 742, 994 and 972 individuals were sampled, respectively^13–15^. The detected level of pneumococcal colonisation remained stable over these winters, with 190 (26% prevalence), 232 (23% prevalence) and 294 (30% prevalence) *S. pneumoniae* isolates recovered in the three successive sampling periods. This collection has been used to analyse the changing antigenic profile of the population in response to vaccine-induced immunity through serological typing^13–15^. The same collection was also genotyped by multilocus sequence typing^16^ (MLST) to relate these changes to the elimination, emergence and diversification of individual bacterial lineages^17,18^. Subsequently, whole genome sequencing was applied to the collection to investigate the population dynamics in greater detail^19^. This dataset consists of 616 annotated draft *S. pneumoniae* genomes representing the evolutionary epidemiology of the species as the population structure changed in response to vaccine-induced selection pressures. To aid analysis of the overall set of isolates, the species-wide core genome alignment and phylogeny are also made available, as are the predicted protein sequences, a method for inferring serotype from Illumina reads, and whole genome alignments for fifteen sets of closely-related isolates.

## Methods

### Culturing and phenotyping of strains

Following retrieval from storage, all bacterial samples were colony purified, then grown on 5% sheep’s blood agar overnight at 37 °C in the presence of 5% CO_2_. Samples were serologically typed using latex agglutination (Statens Serum Institut, Copenhagen) as a check on sample handling. Discrepant results with previous typing were verified using the Quellung reaction (Statens Serum Institut, Copenhagen; Table 1 (available online only)).

Overnight plate growth was harvested through resuspension in phosphate buffered saline, and genomic DNA was extracted using DNeasy columns (Qiagen) following manufacturer’s instructions. The concentration of genomic DNA was quantified using the Qubit system (Life Technologies); all samples yielded at least 3 μg of DNA. The integrity of the genomic DNA was checked using agarose gel electrophoresis relative to a λHindIII ladder (New England Biolabs).

### Generation of sequence data

Illumina sequencing libraries were constructed as described previously^20,21^. Briefly, genomic DNA was first fragmented using Adaptive Focused Acoustics technology (Covaris). The resulting fragments were then repaired to ensure they had blunt ends, phosphorylated at their 5′ end, A-tailed at the 3′ end, and ligated to adapter molecules. This library of fragments was then separated by agarose gel electrophoresis. DNA constructs of the appropriate size range (generating an insert size of approximately 150–300 bp) were then extracted from the gel and amplified by a polymerase chain reaction using Kapa HiFi polymerase (Kapa Biosystems) that added one of the 96 index tags used in this project. Libraries were then quantified using qPCR, and combined into an equimolar pool of 96 samples prior to denaturation, cluster generation and sequencing in a single flow cell lane with an Illumina HiSeq machine. Isolates from 2001 and 2003–2004 were sequenced as paired end libraries generating 75 nt reads; isolates from 2006–2007 were sequenced as paired end libraries generating 100 nt reads.

### Assembly and annotation of sequence data

Sequences were assembled *de novo* using Velvet^22^ version 1.2 with parameters selected to be optimal for individual datasets as described previously^23^. Both Glimmer3 (ref. 24) and Prodigal^25^ were trained on the reference sequence of *S. pneumoniae* ATCC 700669 (ref. 26; Data Citation 1), then applied to the complete draft assembly with an 11 nt sequence encoding stop codons in each reading frame added to each end, to facilitate the identification of partial coding sequences (CDSs) broken by the assembly. Putative CDSs were then trimmed at the 3′ end to stop them spanning contig breaks within the assembly. Final CDS predictions were identified as the consensus of Glimmer3 and Prodigal outputs, as described previously^19^ (Table 1 (available online only)). Protein sequences were then translated, aligned using BLAT^27^ suite 0.34, and ‘clusters of orthologous genes’ (COGs) identified using COGsoft^28^. Pairs of orthologous sequences were then manually identified as described previously^19^.

To generate functional annotations of genome sequences, all CDSs were labelled with a unique identifier (of the form, ‘ERSX_Y’, where ‘ERSX’ is the sample accession code in the European Nucleotide Archive listed in Table 1 (available online only) and Y is an incrementing index) and their COG (of the form, ‘SPARC1_CLSZ’ or ‘SPARC1_CLSTZ’, where Z is a number). COGs relating to antibiotic resistance and the newly-characterised variable restriction-modification system loci were annotated as described previously^29^; the 590 COGs found to be specific to prophage, the three COGs found to be specific to a particular prophage remnant, the 142 COGs found to be specific to phage-related chromosomal islands, and the 355 COGs found to be specific to integrative and conjugative elements were also appropriately identified in these datasets^29^. All COGs not belonging to one of these categories were annotated using a database of pneumococcal CDS information. This was constructed by extracting the protein sequences and annotated functions from publicly-available complete genomes and the annotation of 90 capsule polysaccharide synthesis loci^30^. Where a CDS in one of the draft genomes had a putative protein sequence identical to the translated sequence of a previously annotated locus, the annotation was directly transferred; otherwise, the annotation was transferred on the basis of orthology, if another putative protein in the same COG was identical to the translation of a CDS in an annotated genome sequence. In cases where no such information could be obtained, CDSs were labelled as producing ‘hypothetical proteins’. Pneumococcal small interspersed repeats were annotated as described previously^31^, and tRNA and rRNA loci were annotated with tRNAscan-SE^32^ version 1.3.1 and rnammer^33^ version 1.2, respectively (Table 1 (available online only)).

### Generation of core genome alignment and overall phylogeny

As described previously^19^, the 1,194 COGs found to have a single representative in each of the 616 genomes were individually aligned at the protein level using MUSCLE^34^, prior to backtranslation to generate a 1.14 Mb codon alignment. The 106,196 polymorphic sites were extracted and used to generate a phylogeny using RAxML^35^ version 7.0.4 with the general time reversible substitution model and a four category gamma distribution to account for rate heterogeneity. This tree was midpoint rooted on the longest branch, which separated sequence cluster 12 from the rest of the population. This is consistent with a wider phylogenetic analysis of multiple species that suggested sequence cluster 12 was the earliest lineage to diverge from the other isolates^19^. The same alignment was analysed with BAPS^36^ version 5 to identify the sequence clusters. Both the core genome alignment and tree are made available as part of Data Citation 2.

### Generation of whole genome alignments

For each of the fifteen monophyletic sequence clusters identified using BAPS and RAxML, a single reference draft assembly was selected for manual curation. The Illumina read data were reassembled with SGA^37^ version 0.9, and these contigs merged with those from Velvet using Zorro^38^ version 2.2. These were arranged into scaffolds using SSPACE^39^ version 2, then manually curated and ordered using ABACAS^40^ and ACT^41^. These fifteen assemblies are made available as part of Data Citation 2. Illumina read pairs from isolates of the same sequence cluster were then mapped against this reference using SMALT^42^ version 0.5.8. The resulting read alignment was processed with Samtools^43^, VCFtools^44^ and Biopython^45^ to generate a consensus sequence. Bases were called at positions spanned by at least two reads in each direction, where at least a 75% consensus on the allele was evident; additionally, the base quality at the site had to be at least 50, and the mapping quality had to be at least 30, on the Phred scale^46,47^. These consensus sequences from each representative of the sequence cluster were then combined to generate a reference-based multiple genome alignment, each of which was analysed as described previously using an earlier version of the Gubbins^48^ software. These fifteen whole genome alignments, which do not include the reference assemblies themselves, are also made available as part of Data Citation 2.

## Code Availability

The algorithm used to predict recombination events has been developed into a software package, named Gubbins^48^, which can be installed on Linux and Max OSX operating systems. It can also be run on Windows operating systems using a virtual machine environment, and is freely available from http://sanger-pathogens.github.io/gubbins/. Code and reference sequences for the serological typing of *S. pneumoniae* using sequence reads is made available as part of Data Citation 2.

## Data Records

The raw sequence data (FASTQ format) and annotated draft genome sequences (EMBL format) for the 616 isolates in the dataset have been deposited in the European Nucleotide Archive (http://www.ebi.ac.uk/ena/) with the project accession code in Data Citation 3. Individual accession codes for both raw read data and annotated draft genome assemblies are listed in Table 1 (available online only) and in the machine-readable ISA-tab metadata files associated with this article.

Further files are made available through the Dryad repository (https://datadryad.org) with the digital object identifier in Data Citation 2. The core genome codon alignment (FASTA format) and maximum likelihood phylogeny (Newick format) describe the overall population structure. For the study of individual lineages, a whole genome alignment (FASTA format) and draft reference assembly (FASTA format) are included for each of the 15 monophyletic sequence clusters^19^. These encompass 491 of the isolates, excluding those in the diverse polyphyletic sequence cluster 16. In addition, the full set of protein coding sequences (FASTA format), and the translated proteins (FASTA format), can be used to study the diversity of individual COGs. To minimise the manipulation of the protein sequences, the initial amino acid of each protein is directly translated, rather than being converted to a methionine.

The epidemiological and phylogenetic data can also be interactively visualised and analysed online using the Microreact website (http://microreact.org/) with the URL in Data Citation 4.

## Technical Validation

### Integrity of sample handling and quality control

**Figure 1:**
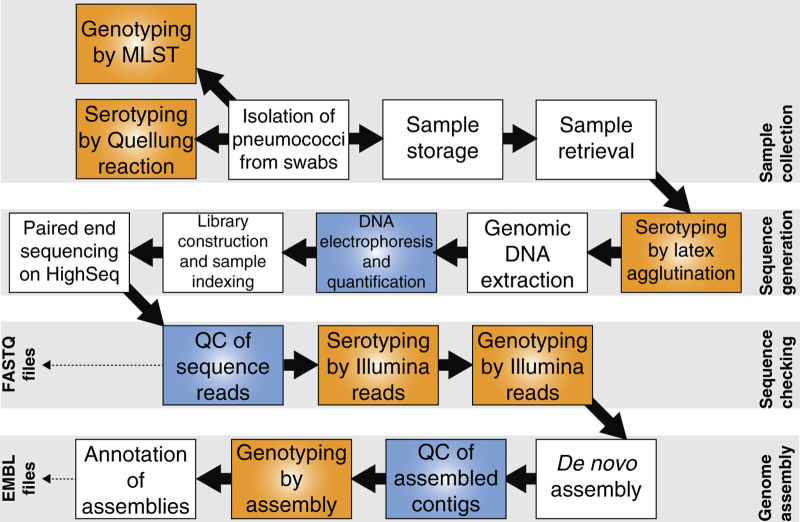
Workflow for the generation of draft genome assemblies. The flow chart shows the steps taken to generate the draft genome dataset; boxes in orange indicate steps at which typing was performed, allowing the integrity of sample handling to be checked, and boxes in blue indicate steps at which checks were performed to allow for the identification and elimination of low quality samples.

An overview of the processing pipeline, including the technical validation steps, is shown in Fig. 1. Of the 716 samples collected as part of the surveillance project between 2001 and 2007, 631 could be revived and cultured. All these isolates were subjected to serological typing using latex agglutination to ensure consistency of sample handling relative to previous studies (Fig. 1 and Table 1 (available online only)). As multiple colonisation is often observed in children^49^ it was not necessarily expected that the original strain would be recovered in all cases; in the earlier studies, only a single isolate per individual was analysed in order to maximise the size of the host population sample, as detecting strains carried at low frequencies is inefficient using standard microbiological techniques^50^. The serology differed from that previously recorded for 12 of the 631 samples. These new serotype inferences were all independently verified as being correct using a second culture of the same isolate, and subsequently confirmed in all seven cases where the samples were retyped in a separate laboratory using the Quellung reaction^51^. One sample yielded two clearly morphologically distinct strains that proved to be of different serotypes, increasing the number of isolates in the study to 632. All isolates found to be non-typeable serologically were tested for optochin susceptibility using ‘P discs’; this identified one isolate as likely to represent a non-pneumococcal streptococcus, the exclusion of which reduced the overall collection size to 631.

Sufficient high-quality genomic DNA for analysis was extracted from all isolates. After sequencing, nine samples failed either automated quality control checks implemented by the Sanger Institute, or manual investigation of dataset properties, such as adapter content, insert size and GC content; one of these failures was successfully resequenced. After assembly of the remaining 623 samples, seven further samples were rejected as generating low quality draft genomes. These represented cases where the N50 was below 15 kb and the total assembly length was greater than 2.4 Mb, likely as a consequence of sequence from more than one isolate being mixed in the raw Illumina reads. This resulted in the final dataset of 616 draft genomes.

### Integrity of data handling

**Figure 2:**
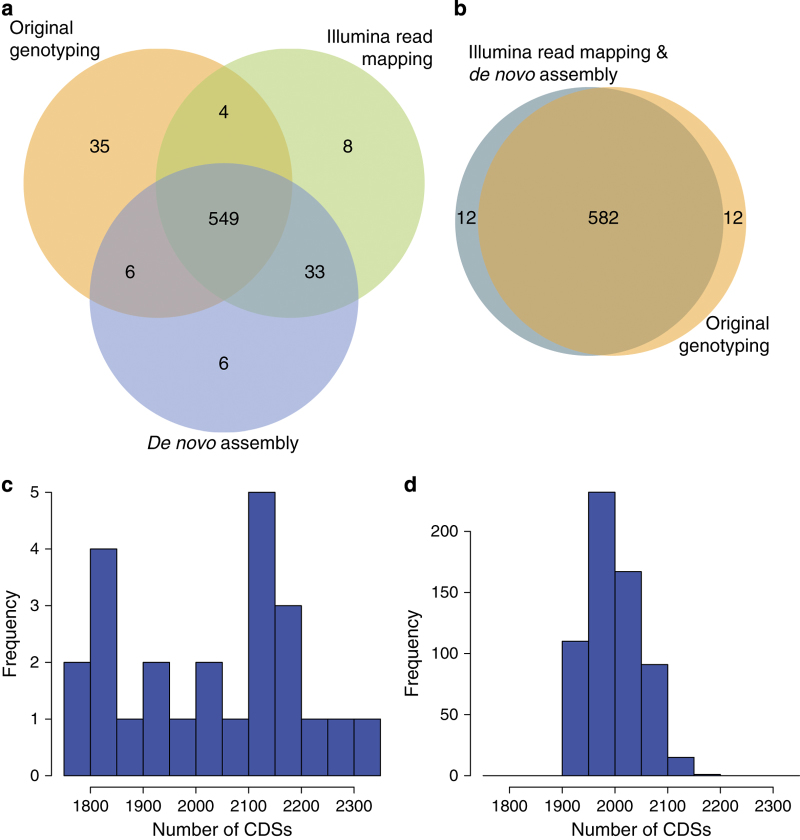
Validation of the draft genome assemblies. (**a**) Venn diagram showing the overlap between the sequence types from the original genotyping of the collection, those inferred from Illumina sequence read mapping, and those inferred from the genome assemblies. Only data for the 594 isolates for which all three datatypes were available are represented here. (**b**) Venn diagram showing the overlap between sequence types inferred by different methodologies, in this case treating results as being consistent if only one of the seven loci differed between results. In this case, the sequence types inferred from read mapping and *de novo* assembly are identical, and differ from the original genotyping in only twelve cases. (**c**) Histogram showing the number of CDSs in publicly available annotated complete, or high quality draft, *S. pneumoniae* genomes. (**d**) Histogram showing the number of CDSs in the 616 draft genomes from Massachusetts. This distribution shows that the count of putative CDSs in each draft genome is within the range of CDSs identified in manually annotated genomes, consistent with the draft genomes being near-complete, and the CDS predictions being accurate.

Serotypes and multilocus sequence types (STs) were inferred from Illumina read data as described previously^23^. Excluding isolates determined as being ‘non typeable’ by either microbiological or bioinformatic serotyping, across the final set of 616 samples the capsule polysaccharide synthesis (*cps*) locus was congruent with the serogroup (a set of antigenically similar serotypes) identified through immunological tests in all but two cases. Of the 594 isolates for which an ST had been previously established, 553 (93%) were identical with those inferred from Illumina reads (Fig. 2a). These included both cases where the genome’s *cps* locus did not match the experimentally ascertained serogroup, indicating these discrepancies were not likely to result from sampling handling issues. Of the 41 cases in which the original ST was discrepant with that inferred from the reads, 29 differed at only one of the seven loci (Fig. 2b). All remaining inconsistencies are likely to reflect instances of multiple colonisation, resulting in different strains being originally genotyped before storage and subsequently retrieved for sequencing. This is consistent with the *cps* locus from the sequence reads in these cases matching the serology of the revived isolates from which the genomic DNA was extracted.

### Quality of genome assemblies

The 616 samples in the dataset each yielded between 267 and 1,865 Mb of sequence data (median of 652 Mb). Assuming a typical 2 Mb *S. pneumoniae* genome^26^, this meant each isolate had a sequencing depth of over 100 fold coverage. Based on a random sample of 10,000 reads aligned to a set of prokaryotic and eukaryotic reference genomes using BWA^52^, a substantial majority of reads matched to the *S. pneumoniae* representative (strain ATCC 700669) in each dataset, confirming these data were primarily derived from the submitted genomic DNA sample.

All draft assemblies had an N50 greater than 15 kb and a total length between 1.98 and 2.19 Mb, similar to complete *S. pneumoniae* genomes. Additionally, the number of CDSs in the *de novo* assemblies was within the range of CDSs found within annotated complete or high-quality draft *S. pneumoniae* genomes (Fig. 2c,d). Each isolate’s annotation included at least 101 of the 102 protein functional domains recently suggested to be essential and ubiquitous across cellular genomes^53^; the only discrepancy was the short ribosomal protein coding sequence *rpsN*, which was not consistently identified by the automated gene annotation software even when present within assemblies. Assembly quality was also judged on the basis of non-coding RNA content: using previously-defined criteria^53^, the majority of isolates had a full-length representative of each of the ribosomal RNAs. Similarly, quantifying tRNA content found the majority of isolates had at least one tRNA for each standard amino acid.

To ascertain the accuracy of the *de novo* assemblies relative to the original epidemiological data and Illumina sequence reads, the STs were extracted from the contigs through identification of the relevant loci using BLAST^54^ (Fig. 2a,b). All seven loci could be recovered from each assembly. Across the 616 samples, the ST extracted from the assembly and Illumina reads was identical in 602 cases (98% accuracy). In four cases, the ST inferred from the assemblies differed from the consensus of the original genotyping and the ST extracted from the reads at a single locus (Fig. 2a). In six cases, the ST inferred from the Illumina reads differed from the consensus of the original genotyping and the assembly at a single locus; the assemblies indicated these all corresponded to a single ST, suggesting a rare systematic error. In four cases, the STs inferred from the assembly and Illumina reads differed at a single locus, and the original genotyping data were missing or inconsistent with both.

**Figure 3:**
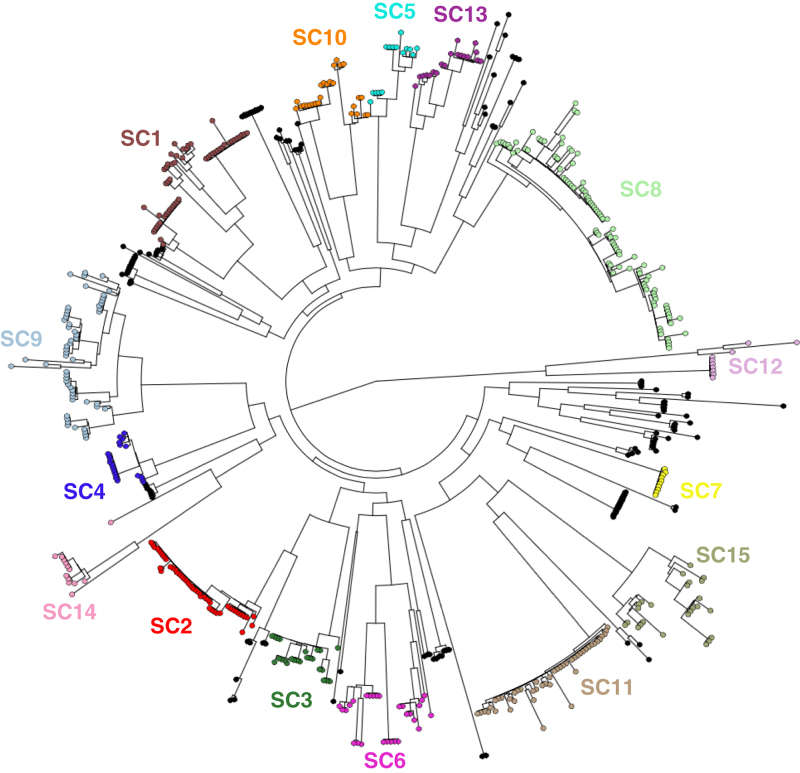
Overall population structure of the 616 *S. pneumoniae* isolates. The maximum likelihood phylogeny generated from the core genome alignment is presented, as displayed in the Microreact system (Data Citation 4), with each leaf node coloured according to its sequence cluster (SC).

The wider ‘core’ genome was defined as a set of 1,194 COGs, a single representative of which was found in each genome^19^. Independent re-analysis of this dataset with a different method for defining COGs found 1,206 ‘core’ COGs, of which 1,027 were identical to the 1,194 originally identified^55^. Concatenated codon alignments of the ‘core’ COGs were subject to three independent analyses with BAPS version 5, which converged on identical membership of the sixteen sequence clusters, in two cases, with additional isolates included within SC7 in the third. The fifteen sequence clusters containing similar isolates were correspondingly monophyletic in the core genome phylogeny, confirming them as being closely related sets of bacteria (Fig. 3).

### Validation of recombination analyses

Recombination detection was only attempted within the 15 monophyletic sequence clusters, as they consisted of groups of isolates with detectable similarity that was likely to reflect recent common ancestry. Simulations indicated that the type of approach that was used to identify recombinations in whole genome alignments is most accurate when applied to sets of closely-related sequences^48^. In the analyses presented in this work, all alignments in which at least ten recombination events were detected formed an exponential recombination length distribution with a rate constant consistent with other genomic data^23,56–58^ and experimental work^47^. The positions of recombination ‘hotspots’ relative to the reference genome annotations were also consistent with these independent analyses. In the cases where evidence of a molecular clock could be detected, the substitution rate was also found to be consistent with the analyses of other *S. pneumoniae* datasets^23,56–58^. Additionally, the consistency of the final phylogenies with the epidemiological data allowed a phylogeographic signal to be detected^19^.

Further analysis of the isolates’ inferred serology (Table 1 (available online only)) identified cases where closely-related isolates differed at their *cps* loci, suggesting ‘serotype switching’ had occurred. In all cases where the pattern of switching could be robustly established, the change at the *cps* locus could be attributed to an inferred recombination affecting the relevant genes^4^.

## Usage Notes

Sequence data may be downloaded from the European Nucleotide Archive using the project accession codes ERP000809 or PRJEB2632. All accession codes for raw sequence data and annotated individual assemblies are listed in Table 1 (available online only). Associated epidemiological data was published previously^19^. Sequences and functional annotation can be displayed using Artemis^41^. Whole genome alignments can be viewed and analysed using standard software. Gubbins^48^ can be applied to them for the inference of recombined sequence. The software for serological typing can be run on Linux or Mac OSX as described in the accompanying README file.

## Additional Information

Tables 1 is only available in the online version of this paper.

**How to cite this article**: Croucher, N. J. *et al.* Population genomic datasets describing the post-vaccine evolutionary epidemiology of *Streptococcus pneumoniae*. Sci. Data 2:150058 doi: 10.1038/sdata.2015.58 (2015).

## Supplementary Material



## Figures and Tables

**Table 1:** Sample handling integrity and assembly properties

**Isolate Identification**		**Sequence Access**				**Serology**			**Genotyping**			**Genomics**	**Assembly & Annotation Information**					
**Isolate Name**	**Taxon ID**	**Sample Accession**	**Sequence Reads Accession**	**First Contig Annotation Accession**	**Final Contig Annotation Accession**	**Original Serotype**	**Retyping Results (if inconsistent)**	**Serotype From Reads**	**Original Sequence Type**	**Sequence Type From Reads**	**Sequence Type From Assembly**	**Sequence Cluster**	**Total Sequencing Yield (kb)**	**Number of Contigs**	**Assembly N50**	**Number of Coding Sequences**	**Number of tRNAs**	**Number of rRNAs**
1334	001334	ERS044037	ERR069731	CNST02000001	CNST02000153	10		10A	585	585	585	1	722874	153	62775	2000	45	3
279388	279388	ERS044115	ERR069809	CNUR02000001	CNUR02000219		10	10A		97	97	1	695458	219	43531	1998	38	3
CH2079	5Z52R	ERS069979	ERR129088	COCE02000001	COCE02000187	10A		10A	816	816	816	1	553408	187	52304	1973	36	3
LE4000	N5O68	ERS070017	ERR129126	CFKD02000001	CFKD02000152	10A		10A	3290	3290	3290	1	577924	152	54788	1963	35	3
LE4124	RUJ90	ERS070049	ERR129158	COEA02000001	COEA02000141	10A		10A	816	816	816	1	521438	141	77373	1976	45	3
MD5021	29ORI	ERS070055	ERR129164	COEG02000001	COEG02000169	10A		10A	816	816	816	1	537572	169	55810	2024	43	3
ND6034	RN3X8	ERS070090	ERR129199	COFG02000001	COFG02000132	10A		10A	816	816	816	1	539173	132	69832	1972	43	3
ND6039	PKMM4	ERS070092	ERR129201	CFRQ02000001	CFRQ02000122	10A		10A	97	97	97	1	528497	122	82155	1998	45	3
NP7052	N6I98	ERS070147	ERR124256	CNYN02000001	CNYN02000247	10A		10A	97	97	97	1	904732	247	45498	2042	41	3
PT8016	G2Z0B	ERS070176	ERR124285	CNZL02000001	CNZL02000178	10A		10A	461	461	461	1	885713	178	48227	1943	44	3
R34-3021	R34-3021	ERS043842	ERR065347	CNPI02000001	CNPI02000197	10A		10A	585	585	585	1	675631	197	62531	1995	41	3
R34-3024	R34-3024	ERS043845	ERR065350	CNPE02000001	CNPE02000138	10A		10A	585	585	585	1	611325	138	62775	2000	43	3
R34-3111	R34-3111	ERS043926	ERR068012	CNWY02000001	CNWY02000163	10A		10A	816	816	816	1	636208	163	81514	1966	45	3
R34-3157	R34-3157	ERS043962	ERR068048	CNXN02000001	CNXN02000214	10A		10A	97	97	97	1	584492	214	62739	2012	38	3
R34-3182	R34-3182	ERS043987	ERR067981	CFID02000001	CFID02000204	10A		10A	816	816	816	1	619369	204	54789	1970	41	3
R34-3184	R34-3184	ERS043989	ERR069683	CNQQ02000001	CNQQ02000274	10A		10A	816	816	816	1	815231	274	34534	1968	31	3
127530	127530	ERS044074	ERR069768	CNTH02000001	CNTH02000239	35F		35F	1953	1953	1953	1	870022	239	44672	1941	31	3
473937	473937	ERS044165	ERR065968	CNPR02000001	CNPR02000272	35F		35F	498	498	498	1	1627916	272	33037	1947	41	2
BR1082	7C7G1	ERS069942	ERR129051	COAX02000001	COAX02000151	35F		35F	498	498	498	1	498464	151	51813	1952	45	3
BR1104	0CNYC	ERS069949	ERR129058	CFLT02000001	CFLT02000144	35F		35F	498	498	498	1	467092	144	65135	1958	42	3
CH2084	NIPQY	ERS069981	ERR129090	COCF02000001	COCF02000219	35F		35F	498	498	498	1	542021	219	39828	1950	36	3
GL3040	Q7F26	ERS070004	ERR129113	COCX02000001	COCX02000129	35F		35F	498	498	498	1	622745	129	62838	1956	45	3
LE4019	4D0HZ	ERS070023	ERR129132	CODD02000001	CODD02000167	35F		35F	498	498	498	1	637013	167	54225	1957	41	3
ND6142	4HUA7	ERS070128	ERR124237	CNYH02000001	CNYH02000190	35F		35F	498	498	498	1	1478239	190	43543	1947	42	3
NP7076	BL0SN	ERS070156	ERR124265	CHWY02000001	CHWY02000343	35F		35F	498	498	498	1	755247	343	39673	1971	37	3
PT8011	B50V6	ERS070173	ERR124282	CHWZ02000001	CHWZ02000173	35F		35F	498	498	498	1	990352	173	46695	1945	43	3
PT8076	UVVWA	ERS070195	ERR124304	CNZX02000001	CNZX02000184	35F		35F	498	498	498	1	1136340	184	49776	1944	43	3
R34-3112	R34-3112	ERS043927	ERR068013	CHBO02000001	CHBO02000188	35F		35F	498	498	498	1	559483	188	54203	1947	41	3
R34-3118	R34-3118	ERS043932	ERR068018	CNWV02000001	CNWV02000141	35F		35F	498	498	498	1	711343	141	65342	1950	45	3
R34-3120	R34-3120	ERS043934	ERR068020	CNWL02000001	CNWL02000156	35F		35F	498	498	498	1	616726	156	70102	1956	45	3
R34-3178	R34-3178	ERS043983	ERR067977	CNQJ02000001	CNQJ02000235	35F		35F	1916	498	498	1	598635	235	44525	1942	36	3
R34-3191	R34-3191	ERS043996	ERR069690	CNRL02000001	CNRL02000228	35F		35F	498	498	498	1	775377	228	44508	2010	43	3
R34-3213	R34-3213	ERS044018	ERR069712	CNSB02000001	CNSB02000158	35F		35F	498	498	498	1	717789	158	70164	2002	42	3
R34-3216	R34-3216	ERS044021	ERR069715	CNRO02000001	CNRO02000152	35F		35F	498	498	498	1	757343	152	70164	1958	45	3
1996	001996	ERS044038	ERR069732	CNSW02000001	CNSW02000164	6A		6A	1360	1360	1360	1	662636	164	54237	2073	45	3
42397	042397	ERS044046	ERR069740	CGDZ02000001	CGDZ02000162	6A		6A	460	460	460	1	828155	162	63702	2065	45	3
62031	062031	ERS044052	ERR069746	CNSM02000001	CNSM02000213	6A		6A	460	460	460	1	801121	213	45193	2055	42	3
89848	089848	ERS044061	ERR069755	CNTP02000001	CNTP02000211	6A		6A	460	460	460	1	763020	211	57007	2115	46	3
118039	118039	ERS044071	ERR069765	CNTJ02000001	CNTJ02000174	6A		6A	460	460	460	1	847182	174	69053	2061	45	3
135631	135631	ERS044076	ERR069770	CNTG02000001	CNTG02000149	6A		6A		460	460	1	787420	149	60052	2054	45	3
299801	299801	ERS044118	ERR069812	CNUU02000001	CNUU02000137	6A		6A		460	460	1	653068	137	63718	2062	45	3
336850	336850	ERS044129	ERR069823	CIDR02000001	CIDR02000147	6A		6A	460	460	460	1	709263	147	54381	2054	45	3
383739	383739	ERS044141	ERR069835	CNVJ02000001	CNVJ02000145	6A		6A	460	460	460	1	565432	145	69053	2064	45	3
446376	446376	ERS044159	ERR065962	CFLN02000001	CFLN02000324	6A		6A	460	460	460	1	1222840	324	24765	2045	31	3
472699	472699	ERS044164	ERR065967	CNPJ02000001	CNPJ02000188	6A		6A	460	460	460	1	1370061	188	63117	2058	41	3
ND6134	CV6JA	ERS070122	ERR124231	CHIS02000001	CHIS02000152	6A		6A	460	460	460	1	824059	152	49835	2050	42	3
R34-3018	R34-3018	ERS043839	ERR065344	CNPA02000001	CNPA02000199	6A		6A	460	460	460	1	406244	199	49705	2056	41	3
R34-3035	R34-3035	ERS043856	ERR065292	CNNG02000001	CNNG02000193	6A		6A	460	460	460	1	732888	193	49705	2058	43	3
R34-3075	R34-3075	ERS043894	ERR065330	CNOT02000001	CNOT02000144	6A		6A	460	460	460	1	476229	144	56775	2061	45	3
R34-3166	R34-3166	ERS043970	ERR067964	CNQX02000001	CNQX02000142	6A		6A	460	460	460	1	560872	142	57073	2039	45	3
R34-3220	R34-3220	ERS044025	ERR069719	CNRU02000001	CNRU02000142	6A		6A	1919	460	460	1	754252	142	57223	2064	45	3
R34-3222	R34-3222	ERS044027	ERR069721	CFQO02000001	CFQO02000146	6A		6A	460	460	460	1	829670	146	69245	2057	45	3
74124	074124	ERS044058	ERR069752	CFJS02000001	CFJS02000112	11A		11D	1913	1913	1913	2	859799	112	73726	1925	44	3
102720	102720	ERS044066	ERR069760	CNTQ02000001	CNTQ02000113	11A		11D		62	62	2	750419	113	70740	1947	45	3
242588	242588	ERS044107	ERR069801	CNUT02000001	CNUT02000118	11A		11D		62	62	2	659925	118	61941	1944	45	3
263137	263137	ERS044110	ERR069804	CNVC02000001	CNVC02000109	11A		11D	62	62	62	2	570680	109	68782	1938	45	3
335574	335574	ERS044128	ERR069822	CNVD02000001	CNVD02000108	11A		11D	62	62	62	2	612642	108	64773	1939	46	3
386329	386329	ERS044143	ERR069837	CNVM02000001	CNVM02000123	11A		11D	1913	1913	1913	2	732342	123	70789	1927	34	3
389109	389109	ERS044145	ERR069839	CNVL02000001	CNVL02000162	11A		11D	62	62	62	2	724678	162	56861	1949	36	3
459747	459747	ERS044161	ERR065964	CNPH02000001	CNPH02000161	11A		11D	62	62	62	2	1718828	161	53562	1985	46	3
BR1039	FP9DM	ERS069928	ERR129037	COAM02000001	COAM02000109	11A		11D	62	62	62	2	459396	109	64879	1937	43	3
BR1117	E4NSI	ERS069959	ERR129068	COBM02000001	COBM02000109	11A		11D	62	62	62	2	500706	109	65403	1928	46	3
CH2053	DFS2Q	ERS069970	ERR129079	COBY02000001	COBY02000151	11A		11D	62	62	62	2	519374	151	49076	1932	41	3
CH2054	K9N0O	ERS069971	ERR129080	COCA02000001	COCA02000155	11A		11D	3276	3276	3276	2	535340	155	48753	1925	38	3
CH2091	HWJVH	ERS069984	ERR129093	CFKA02000001	CFKA02000119	11A		11D	62	62	62	2	608909	119	59135	1940	45	3
LE4005	R52DJ	ERS070018	ERR129127	CFLV02000001	CFLV02000116	11A		11D	62	62	62	2	557016	116	64405	1943	46	3
LE4018	6ZCAZ	ERS070022	ERR129131	CODG02000001	CODG02000110	11A		11D	66	62	62	2	698664	110	64405	1939	45	3
LE4040	H3Q9M	ERS070028	ERR129137	CHBV02000001	CHBV02000106	11A		11D	3291	62	62	2	530145	106	68768	2001	47	3
LE4050	J1LX6	ERS070030	ERR129139	CFRE02000001	CFRE02000118	11A		11D	62	62	62	2	513346	118	63765	1941	46	3
LE4111	F1G5R	ERS070045	ERR129154	CODX02000001	CODX02000093	11A		11D	62	62	62	2	442076	93	77111	1929	46	3
LE4127	F1ODP	ERS070050	ERR129159	COEB02000001	COEB02000099	11A		11D	62	62	62	2	449472	99	68793	1986	46	3
MD5070	9ABKR	ERS070068	ERR129177	COEO02000001	COEO02000107	11A		11D	62	62	62	2	503807	107	64885	1935	47	3
MD5071	8AH8E	ERS070069	ERR129178	COES02000001	COES02000109	11A		11D	62	62	62	2	569828	109	65448	1934	46	3
ND6073	5J97G	ERS070101	ERR129210	COFS02000001	COFS02000108	11A		11D	62	62	62	2	816213	108	58324	1924	47	3
ND6085	3YFA4	ERS070102	ERR129211	COFT02000001	COFT02000125	11A		11D	62	62	62	2	551204	125	64405	1994	46	3
ND6103	KYDZM	ERS070105	ERR129214	COFW02000001	COFW02000112	11A		11D	62	62	62	2	506942	112	63950	1943	46	3
ND6104	QHXZZ	ERS070106	ERR129215	COFV02000001	COFV02000120	11A		11D	62	62	62	2	579315	120	64405	1975	46	3
ND6151	8G3XE	ERS070131	ERR124240	CNYB02000001	CNYB02000193	11A		11D	62	62	62	2	1202172	193	41072	1932	31	3
ND6156	T14BF	ERS070133	ERR124242	CNYC02000001	CNYC02000112	11A		11D	62	62	62	2	1012160	112	58325	1933	45	3
NP7010	F7IXH	ERS070137	ERR124246	CNYK02000001	CNYK02000108	11A		11D	62	62	62	2	1313562	108	59102	1933	47	3
NP7095	RAV85	ERS070159	ERR124268	CFJT02000001	CFJT02000116	11A		11D	62	62	62	2	663964	116	64165	1929	46	3
PT8037	RQ6NH	ERS070182	ERR124291	CNZP02000001	CNZP02000109	16F	11	11D	62	62	62	2	825421	109	59058	1933	42	3
PT8053	6CO3C	ERS070187	ERR124296	CNZR02000001	CNZR02000140	11A		11D	62	62	62	2	1507531	140	55698	1915	36	3
PT8063	XCJ97	ERS070189	ERR124298	CNZQ02000001	CNZQ02000219	11A		11D	62	62	62	2	1865382	219	49577	1993	42	2
PT8072	28I7H	ERS070193	ERR124302	CNZW02000001	CNZW02000104	11A		11D	62	62	62	2	693076	104	70414	1980	47	3
R34-3052	R34-3052	ERS043872	ERR065308	CNNY02000001	CNNY02000119	11A		11D	62	62	62	2	663300	119	68923	1944	45	3
R34-3054	R34-3054	ERS043874	ERR065310	CNOA02000001	CNOA02000104	11A		11D	62	62	62	2	506385	104	72751	1933	46	3
R34-3071	R34-3071	ERS043890	ERR065326	CHBF02000001	CHBF02000117	11A		11D	62	62	62	2	480823	117	71626	1983	46	3
R34-3080	R34-3080	ERS043899	ERR067985	CFRA02000001	CFRA02000116	11A		11D	62	62	62	2	797111	116	64720	1931	46	3
R34-3081	R34-3081	ERS043900	ERR067986	CNWJ02000001	CNWJ02000115	11A		11D	62	62	62	2	781069	115	71626	1938	47	3
R34-3095	R34-3095	ERS043914	ERR068000	CNWE02000001	CNWE02000125	11A		11D	62	62	62	2	673158	125	64778	1959	46	3
R34-3147	R34-3147	ERS043954	ERR068040	CNXD02000001	CNXD02000109	11A		11D	1933	1913	1913	2	563601	109	71626	1933	44	3
R34-3149	R34-3149	ERS043955	ERR068041	CNXF02000001	CNXF02000136	11A		11D	408	408	408	2	580640	136	61830	1973	41	3
R34-3155	R34-3155	ERS043960	ERR068046	CNXL02000001	CNXL02000123	11A		11D	62	62	62	2	700639	123	68173	1948	45	3
R34-3156	R34-3156	ERS043961	ERR068047	CNXJ02000001	CNXJ02000112	11A		11D	62	62	62	2	610537	112	64773	1944	46	3
R34-3170	R34-3170	ERS043974	ERR067968	CNQR02000001	CNQR02000115	11A		11D	62	62	62	2	408141	115	73242	1926	45	3
R34-3203	R34-3203	ERS044008	ERR069702	CNRI02000001	CNRI02000144	11A		11D	62	62	62	2	769725	144	58857	1993	45	3
R34-3208	R34-3208	ERS044013	ERR069707	CNRX02000001	CNRX02000118	11A		11D	62	62	62	2	809261	118	72751	1975	46	3
R34-3214	R34-3214	ERS044019	ERR069713	CNSC02000001	CNSC02000120	11A		11D	62	62	62	2	860741	120	64773	1932	46	3
R34-3228	R34-3228	ERS044033	ERR069727	CNRZ02000001	CNRZ02000108	11A		11D	62	62	62	2	753887	108	68782	1942	45	3
BR1003	SLXY1	ERS069917	ERR129026	CHIT02000001	CHIT02000165	15A		15A	63	63	63	3	579955	165	64610	2081	43	3
BR1086	IB8MW	ERS069945	ERR129054	COAZ02000001	COAZ02000130	15A		15A	3282	1914	1914	3	524445	130	68635	1982	45	3
BR1106	W5HGR	ERS069951	ERR129060	COBD02000001	COBD02000122	15A		15A	63	63	63	3	512579	122	62192	2051	44	3
BR1109	KRHYA	ERS069952	ERR129061	COBE02000001	COBE02000141	15A		15A	63	63	63	3	424418	141	93521	2067	44	3
ND6028	XCB66	ERS070089	ERR129198	COFH02000001	COFH02000122	15A		15A	63	63	63	3	485959	122	69814	1989	45	3
ND6147	UJECB	ERS070130	ERR124239	CNYF02000001	CNYF02000176	15A		15A	63	63	63	3	1298010	176	64610	1991	38	3
NP7017	CSI7H	ERS070140	ERR124249	CNYO02000001	CNYO02000143	15A		15A	63	63	63	3	951262	143	54713	1980	41	3
NP7105	Z4952	ERS070163	ERR124272	CHIQ02000001	CHIQ02000226	15A		15A	1915	1915	1915	3	1226349	226	56655	2075	42	3
NP7112	XVMDP	ERS070165	ERR124274	CFIT02000001	CFIT02000236	15A		15A	1915	1915	1915	3	891236	236	65314	2075	47	3
PT8013	T8K26	ERS070174	ERR124283	CNZJ02000001	CNZJ02000181	15A		15A	63	63	63	3	875403	181	54231	1974	41	3
PT8065	8QTW4	ERS070191	ERR124300	CFIV02000001	CFIV02000259	15A		15A	63	63	63	3	1696803	259	22962	1968	33	3
R34-3036	R34-3036	ERS043857	ERR065293	CNNQ02000001	CNNQ02000133	15A		15A	1914	1914	1914	3	606950	133	65159	1976	45	3
R34-3040	R34-3040	ERS043861	ERR065297	CNNJ02000001	CNNJ02000132	15A		15A	63	63	63	3	647342	132	61778	2001	45	3
R34-3064	R34-3064	ERS043884	ERR065320	CNOI02000001	CNOI02000150	15A		15A	63	63	63	3	430697	150	70075	2068	46	3
R34-3077	R34-3077	ERS043896	ERR065332	CNOR02000001	CNOR02000147	15A		15A	63	63	63	3	495406	147	64598	1995	41	3
R34-3126	R34-3126	ERS043940	ERR068026	CIDQ02000001	CIDQ02000169	15A		15A	63	63	63	3	602486	169	56726	1977	41	3
R34-3130	R34-3130	ERS043942	ERR068028	CHWX02000001	CHWX02000157	15A		15A	63	63	63	3	694582	157	63156	1979	41	3
R34-3138	R34-3138	ERS043946	ERR068032	CNXP02000001	CNXP02000191	15A		15A	63	63	63	3	694702	191	55610	2058	39	3
R34-3150	R34-3150	ERS043956	ERR068042	CNXE02000001	CNXE02000166		15	15A		1914	1914	3	654017	166	56424	1968	43	3
R34-3158	R34-3158	ERS043963	ERR068049	CNXM02000001	CNXM02000194	15A		15A	63	63	63	3	618935	194	57605	2065	42	3
R34-3179	R34-3179	ERS043984	ERR067978	CNQK02000001	CNQK02000173	15A		15A	1915	1915	1915	3	673246	173	56726	2030	42	3
R34-3225	R34-3225	ERS044030	ERR069724	CNRS02000001	CNRS02000136	15A		15A	63	63	63	3	742785	136	65204	1995	43	3
R34-3226	R34-3226	ERS044031	ERR069725	CHBI02000001	CHBI02000139	15A		15A	63	63	63	3	733637	139	63156	1994	43	3
ND6117	HB7S5	ERS070112	ERR124221	CFIR02000001	CFIR02000119	19A		19A	63	63	63	3	850674	119	58974	2009	45	3
R34-3199	R34-3199	ERS044004	ERR069698	CNRG02000001	CNRG02000154	19A		19A	63	63	63	3	802951	154	54701	2053	45	3
35100	035100	ERS044044	ERR069738	CNSI02000001	CNSI02000135	22F		22F	1901	1901	1901	4	742235	135	56595	2053	46	3
120581	120581	ERS044073	ERR069767	CNTI02000001	CNTI02000163	22F		22F	433	433	433	4	804432	163	46699	1995	43	3
153408	153408	ERS044084	ERR069778	CNUK02000001	CNUK02000130	22F		22F	433	433	433	4	820300	130	55492	2039	48	3
163286	163286	ERS044089	ERR069783	CNUN02000001	CNUN02000129	22F		22F	433	433	433	4	809024	129	53587	1997	47	3
385385	385385	ERS044142	ERR069836	CNVI02000001	CNVI02000134	22F		22F	433	433	433	4	727420	134	56595	2049	48	3
CH2108	RWZJE	ERS069987	ERR129096	COCO02000001	COCO02000121	22F		22F	433	433	433	4	553476	121	57139	1992	47	3
LE4118	FBPNS	ERS070047	ERR129156	CODZ02000001	CODZ02000138	22F		22F	433	433	433	4	496383	138	56232	1990	43	4
ND6047	120T7	ERS070095	ERR129204	COFQ02000001	COFQ02000135	22F		22F	433	433	433	4	266801	135	56753	2004	44	3
NP7013	FJZZW	ERS070139	ERR124248	CNYM02000001	CNYM02000145	22F		22F	433	433	433	4	997588	145	52943	1992	42	3
NP7062	Q3H1U	ERS070151	ERR124260	CFLO02000001	CFLO02000165	22F		22F	1901	1901	1901	4	708360	165	42558	2049	46	3
PT8085	DQKYE	ERS070198	ERR124307	COAA02000001	COAA02000150	22F		22F	433	433	433	4	1155165	150	44870	1994	47	2
PT8116	I0UZQ	ERS070209	ERR124318	COAF02000001	COAF02000109	22F		22F	433	433	433	4	428840	109	50671	1965	45	3
R34-3012	R34-3012	ERS043833	ERR065338	CNOY02000001	CNOY02000144	22F		22F		1337	1337	4	574493	144	61942	2042	42	3
R34-3017	R34-3017	ERS043838	ERR065343	CNPG02000001	CNPG02000134	22F		22F	433	433	433	4	530776	134	61715	2018	45	3
R34-3082	R34-3082	ERS043901	ERR067987	CNWI02000001	CNWI02000136	22F		22F	1337	1337	1337	4	692808	136	59103	2044	46	3
R34-3090	R34-3090	ERS043909	ERR067995	CNVZ02000001	CNVZ02000151	22F		22F	1337	1337	1337	4	687236	151	56964	2047	46	3
R34-3091	R34-3091	ERS043910	ERR067996	CNWA02000001	CNWA02000125	22F		22F	433	433	433	4	678272	125	59334	1967	45	3
R34-3143	R34-3143	ERS043951	ERR068037	CNXQ02000001	CNXQ02000130	22F		22F	433	433	433	4	726946	130	58849	1966	45	3
R34-3167	R34-3167	ERS043971	ERR067965	CFJR02000001	CFJR02000119	22F		22F	433	433	433	4	578393	119	56746	1995	46	3
R34-3187	R34-3187	ERS043992	ERR069686	CNRN02000001	CNRN02000128	22F		22F	433	433	433	4	807317	128	58409	1991	46	3
R34-3189	R34-3189	ERS043994	ERR069688	CNRA02000001	CNRA02000120	22F		22F	433	433	433	4	735648	120	64385	1978	42	3
BR1085	VPPXV	ERS069944	ERR129053	CIKC02000001	CIKC02000133	11A		11A	156	156	156	5	539305	133	57158	2026	48	3
LE4073	3FBXJ	ERS070035	ERR129144	CHBX02000001	CHBX02000258	15B/C		15C	162	162	162	5	665190	258	50011	2146	44	2
MD5103	NJ41D	ERS070073	ERR129182	COEU02000001	COEU02000189	15B/C		15B	3275	3275	3275	5	550506	189	54884	2087	50	3
ND6019	5MZ1D	ERS070087	ERR129196	COFN02000001	COFN02000332	15B/C		15C	162	162	162	5	604860	332	54585	2174	44	3
R34-3109	R34-3109	ERS043924	ERR068010	CHXA02000001	CHXA02000284		15	15B		162	162	5	527501	284	49960	2078	46	3
GL3021	F8P25	ERS069996	ERR129105	COCT02000001	COCT02000141	19A		19A	1925	1925	1925	5	544855	141	73234	2030	48	3
ND6017	XQR0B	ERS070086	ERR129195	COFF02000001	COFF02000150	19A		19A	1925	1925	1925	5	575890	150	59020	2035	48	3
R34-3093	R34-3093	ERS043912	ERR067998	CNWC02000001	CNWC02000171	19A		19A	1925	1925	1925	5	630576	171	84759	1974	45	3
R34-3215	R34-3215	ERS044020	ERR069714	CNSH02000001	CNSH02000166	19A		19A	1925	1925	1925	5	756771	166	80712	2036	44	3
234323	234323	ERS044105	ERR069799	CFQE02000001	CFQE02000139	9A		9V	156	156	156	5	618848	139	79769	2031	49	3
312942	312942	ERS044124	ERR069818	CNVA02000001	CNVA02000142	9A		9V	156	156	156	5	593918	142	76463	2023	48	3
422264	422264	ERS084155	ERR124320	COAH02000001	COAH02000221	9A		9A	156	156	156	5	458625	221	58853	2104	51	3
R34-3016	R34-3016	ERS043837	ERR065342	CHWT02000001	CHWT02000142	9A		9V	156	156	156	5	399840	142	61379	2039	48	3
CH2029	STPDE	ERS069968	ERR129077	COBW02000001	COBW02000214	9V		9V	162	162	162	5	527198	214	46466	2083	48	3
R34-3045	R34-3045	ERS043865	ERR065301	CNNT02000001	CNNT02000129	9V		9V	156	156	156	5	525405	129	80077	2028	49	3
PT8019	HR2T3	ERS070177	ERR124286	CHBT02000001	CHBT02000131	15B/C		15B	199	3280	3280	6	1196963	131	77326	2029	46	3
PT8025	0FQ8K	ERS070179	ERR124288	CIDT02000001	CIDT02000148	15B/C		15C	3280	3280	3280	6	1052080	148	75361	2024	46	3
PT8044	H8YKW	ERS070185	ERR124294	CFQZ02000001	CFQZ02000133	15B/C		17F	3280	3280	3280	6	822160	133	73299	2022	46	3
PT8054	01ZWM	ERS070188	ERR124297	CNZS02000001	CNZS02000178	15B/C		15C	3280	3280	3280	6	1115345	178	63357	2020	39	3
PT8120	BJF60	ERS070210	ERR124319	COAI02000001	COAI02000130	15B/C		15C	3280	3280	3280	6	405752	130	77366	2038	46	3
BR1055	0GB6K	ERS069933	ERR129042	CHXE02000001	CHXE02000137	23A		23A	338	338	338	6	636440	137	68852	1992	44	3
GL3050	OSEY6	ERS070008	ERR129117	COCY02000001	COCY02000132	23A		23A	338	338	338	6	515889	132	73610	2003	44	3
LE4119	DNB90	ERS070048	ERR129157	CODW02000001	CODW02000128	23A		23A	338	338	338	6	408017	128	74023	2057	45	3
MD5029	5XCD5	ERS070058	ERR129167	COEI02000001	COEI02000186	23A		23A	338	338	338	6	525551	186	59110	2106	35	3
MD5068	TV66E	ERS070067	ERR129176	COER02000001	COER02000154	23A		23A	338	338	338	6	502574	154	63937	1998	42	3
ND6100	UNUOJ	ERS070104	ERR129213	COFR02000001	COFR02000152	23A		23A	338	338	338	6	552262	152	70236	2054	42	3
ND6140	28PCJ	ERS070126	ERR124235	CNXX02000001	CNXX02000140	23A		23A	338	338	338	6	855744	140	63800	2014	44	3
PT8086	A12WS	ERS070199	ERR124308	CFRS02000001	CFRS02000138	23A		23A	338	338	338	6	1008797	138	68852	1990	45	3
R34-3047	R34-3047	ERS043867	ERR065303	CFQX02000001	CFQX02000142	23A		23A	338	338	338	6	580649	142	69404	1995	45	3
R34-3123	R34-3123	ERS043937	ERR068023	CFRD02000001	CFRD02000128	23A		23A	338	338	338	6	611026	128	69536	1986	43	3
R34-3209	R34-3209	ERS044014	ERR069708	CFQF02000001	CFQF02000139	23A		23A	338	338	338	6	855583	139	70958	1997	45	3
BR1058	O3452	ERS069934	ERR129043	COAR02000001	COAR02000161	23B		23B	1373	1373	1373	6	550577	161	69549	2107	41	3
ND6124	1LQKE	ERS070118	ERR124227	CFIS02000001	CFIS02000182	23B		23B	1373	1373	1373	6	1567202	182	60867	2104	43	3
R34-3136	R34-3136	ERS043944	ERR068030	CNXK02000001	CNXK02000135	23A		23F	342	342	342	6	718246	135	75703	2060	48	3
153438	153438	ERS044085	ERR069779	CNUE02000001	CNUE02000178	6B		6A	1827	1827	1827	6	746362	178	58069	2017	38	3
174702	174702	ERS044092	ERR069786	CNTT02000001	CNTT02000182	6B		6A	639	639	639	6	839574	182	66518	1978	40	3
223832	223832	ERS044104	ERR069798	CNUJ02000001	CNUJ02000161		6	6A		138	138	6	709724	161	65138	2012	46	3
481643	481643	ERS044166	ERR065969	CFQD02000001	CFQD02000221	6B		6A	138	138	138	6	1633294	221	42901	2007	41	3
487827	487827	ERS044168	ERR065971	CNPS02000001	CNPS02000180	6B		6A	138	138	138	6	1428334	180	60779	2000	44	3
MD5037	UB6XH	ERS070063	ERR129172	CIIN02000001	CIIN02000160	6B		6A	138	138	138	6	486943	160	64834	2061	45	3
R34-3186	R34-3186	ERS043991	ERR069685	CNRF02000001	CNRF02000174	6B		6A	138	138	138	6	867942	174	59130	2082	45	3
R34-3194	R34-3194	ERS043999	ERR069693	CNRE02000001	CNRE02000165	6B		6A	176	5006	5006	6	789106	165	69823	2053	45	3
MD5030	1TR6C	ERS070059	ERR129168	COEH02000001	COEH02000199	6C		6C	138	138	138	6	648781	199	64315	2077	44	2
BR1116	7RCF0	ERS069958	ERR129067	COBP02000001	COBP02000155	7F		7A	191	191	191	7	645774	155	48777	1922	45	3
CH2008	UFG1A	ERS069964	ERR129073	COBT02000001	COBT02000140	7F		7F	191	191	191	7	513954	140	57733	1929	45	3
ND6120	RJBFJ	ERS070115	ERR124224	CIED02000001	CIED02000153	7F		7A	191	191	191	7	1562436	153	48777	1923	45	3
ND6130	B4R32	ERS070119	ERR124228	CFJV02000001	CFJV02000308	7F		7F	191	191	191	7	973684	308	18795	1912	32	1
ND6158	3BDEM	ERS070134	ERR124243	CFQV02000001	CFQV02000149	7F		7F	191	191	191	7	809421	149	53335	1933	45	3
NP7118	Y89K0	ERS070167	ERR124276	CNZE02000001	CNZE02000145	7F		7F	191	191	191	7	716414	145	53335	1928	45	3
PT8039	D8RPY	ERS070183	ERR124292	CNZU02000001	CNZU02000156	7F		7A	191	191	191	7	968790	156	46612	1922	45	3
PT8094	55NO4	ERS070203	ERR124312	COAB02000001	COAB02000178	7F		7A	191	191	191	7	1303735	178	45214	1925	46	3
R34-3163	R34-3163	ERS043967	ERR067961	CNQM02000001	CNQM02000161	7F		7A	191	191	191	7	537018	161	48765	1920	45	3
R34-3219	R34-3219	ERS044024	ERR069718	CNRQ02000001	CNRQ02000159	7F		7A	191	191	191	7	812469	159	47051	1923	46	3
72782	072782	ERS044057	ERR069751	CNSY02000001	CNSY02000137	15B/C		15C	199	199	199	8	812550	137	69458	1974	45	3
103453	103453	ERS044067	ERR069761	CNSX02000001	CNSX02000168	15B/C		15C	1938	1938	1938	8	696916	168	73491	2024	44	3
146066	146066	ERS044080	ERR069774	CNTK02000001	CNTK02000166	15B/C		15C	199	199	199	8	871621	166	68997	1972	45	2
217475	217475	ERS044101	ERR069795	CHIP02000001	CHIP02000177	15B/C		15C	199	199	199	8	687293	177	67617	1982	38	3
362412	362412	ERS044134	ERR069828	CNVV02000001	CNVV02000225		15	15B		199	199	8	792208	225	36881	1999	35	3
365152	365152	ERS044135	ERR069829	CNVU02000001	CNVU02000166	15B/C		15C		199	199	8	642192	166	82074	1967	45	3
372297	372297	ERS044139	ERR069833	CFLQ02000001	CFLQ02000144	15B/C		15B	199	199	199	8	586284	144	82128	1982	41	3
483391	483391	ERS044167	ERR065970	CNPQ02000001	CNPQ02000152	15B/C		15C	199	199	199	8	1486818	152	66331	1963	45	3
492706	492706	ERS044169	ERR065972	CNPW02000001	CNPW02000175	15B/C		15C	199	199	199	8	1330422	175	54120	1963	41	3
509365	509365	ERS044172	ERR065975	CNPV02000001	CNPV02000205	15B/C		15C	199	199	199	8	1561835	205	58597	2021	41	3
BR1022-2	DH8X5-2	ERS069923	ERR129032	COBQ02000001	COBQ02000160	15B/C		15C	199	199	199	8	595975	160	62867	2021	45	3
BR1023	K51SW	ERS069924	ERR129033	COBS02000001	COBS02000213	15B/C		15C	199	199	199	8	613795	213	69142	2032	42	3
BR1025	PXB8V	ERS069925	ERR129034	CFRH02000001	CFRH02000248	15B/C		15C	3280	199	199	8	483133	248	50861	2018	41	3
BR1068	TG9A8	ERS069937	ERR129046	COAU02000001	COAU02000182	15B/C		15C	199	199	199	8	529229	182	69469	2031	44	3
CH2060	FQPLS	ERS069972	ERR129081	COCC02000001	COCC02000141	15B/C		15C	199	199	199	8	594249	141	75646	1975	44	3
CH2085	WQ9GV	ERS069982	ERR129091	COCH02000001	COCH02000158	15B/C		15C	199	199	199	8	598594	158	64845	1958	41	3
GL3071	IRE4I	ERS070012	ERR129121	CODB02000001	CODB02000154	15B/C		15C	199	199	199	8	523593	154	82175	2032	42	1
GL3080	P4GIC	ERS070016	ERR129125	CFRI02000001	CFRI02000164	15B/C		15C	199	199	199	8	556405	164	81950	2025	43	3
LE4011	EH2WN	ERS070020	ERR129129	COEJ02000001	COEJ02000141	15B/C		15B	199	199	199	8	514322	141	82185	1981	44	3
LE4092	XZHZP	ERS070040	ERR129149	CODR02000001	CODR02000160	15B/C		15C	199	199	199	8	557647	160	62958	2019	44	3
MD5045	AYFPY	ERS070065	ERR129174	COEQ02000001	COEQ02000183	15B/C		15C	3286	199	199	8	568263	183	69028	2008	41	3
MD5052	IMAPY	ERS070066	ERR129175	CHXK02000001	CHXK02000147	15B/C		15C	199	199	199	8	562835	147	84660	2024	45	3
MD5110	QLCSW	ERS070077	ERR129186	CHXG02000001	CHXG02000146	15B/C		15B	199	199	199	8	455340	146	82265	1974	43	3
ND6006	5QBRP	ERS070081	ERR129190	COEY02000001	COEY02000145	15B/C		15B	199	199	199	8	536559	145	68872	1961	45	3
ND6121	PN3IH	ERS070116	ERR124225	CNYY02000001	CNYY02000184	15B/C		15C	199	199	199	8	1254362	184	39433	1977	41	3
ND6132	ESBKM	ERS070120	ERR124229	CNXU02000001	CNXU02000160	15B/C		15C	199	199	199	8	765233	160	82188	2027	41	3
NP7066	PCKMO	ERS070154	ERR124263	CNYV02000001	CNYV02000245	15B/C		15C	199	199	199	8	1350126	245	34161	1974	39	3
NP7081	LJ6JL	ERS070158	ERR124267	CNYZ02000001	CNYZ02000130	15B/C		15B	199	199	199	8	791051	130	67643	1966	42	3
PT8098	IZCEG	ERS070204	ERR124313	COAD02000001	COAD02000140	15B/C		15C	199	199	199	8	500975	140	82185	2018	45	3
R34-3011	R34-3011	ERS043832	ERR065337	CFJQ02000001	CFJQ02000179		15	15B		199	199	8	656273	179	68606	2024	45	3
R34-3014	R34-3014	ERS043835	ERR065340	CNPP02000001	CNPP02000150	15B/C		15B	199	199	199	8	682945	150	68971	1966	45	3
R34-3030	R34-3030	ERS043851	ERR065287	CNNI02000001	CNNI02000162	15B/C		15C	199	199	199	8	651692	162	58594	2018	45	3
R34-3031	R34-3031	ERS043852	ERR065288	CNNP02000001	CNNP02000165	15B/C		15C	199	199	199	8	611548	165	71959	1981	46	3
R34-3034	R34-3034	ERS043855	ERR065291	CNNH02000001	CNNH02000177	15B/C		15C	199	199	199	8	594947	177	58598	2037	46	3
R34-3140	R34-3140	ERS043948	ERR068034	CFJX02000001	CFJX02000182	15B/C		15B	199	199	199	8	650075	182	59385	1970	43	3
R34-3144	R34-3144	ERS043952	ERR068038	CNXB02000001	CNXB02000150	15B/C		15C	199	199	199	8	648996	150	76332	1971	45	3
R34-3153	R34-3153	ERS043958	ERR068044	CNXI02000001	CNXI02000162	15B/C		15C	199	199	199	8	634121	162	68873	1981	43	3
R34-3154	R34-3154	ERS043959	ERR068045	CNXH02000001	CNXH02000167	15B/C		15B	199	199	199	8	597608	167	69008	2007	41	3
R34-3159	R34-3159	ERS043964	ERR068050	CNXO02000001	CNXO02000286	15B/C		15C	1932	1932	1932	8	602138	286	32502	1957	31	3
R34-3160	R34-3160	ERS043966	ERR067960	CNQB02000001	CNQB02000159	15B/C		15C	199	199	199	8	655277	159	66332	1984	45	3
R34-3175	R34-3175	ERS043980	ERR067974	CNQF02000001	CNQF02000189	15B/C		15C	199	199	199	8	686799	189	69088	2027	42	3
R34-3188	R34-3188	ERS043993	ERR069687	CNRM02000001	CNRM02000152	15B/C		15C	199	199	199	8	784459	152	86418	2023	45	3
R34-3192	R34-3192	ERS043997	ERR069691	CNRK02000001	CNRK02000163	15B/C		15C	199	199	199	8	825017	163	56307	1988	45	3
R34-3205	R34-3205	ERS044010	ERR069704	CNQU02000001	CNQU02000156	15B/C		15C	199	199	199	8	785495	156	76350	1956	45	3
R34-3206	R34-3206	ERS044011	ERR069705	CNRB02000001	CNRB02000157	15B/C		15C	199	199	199	8	728920	157	82190	1951	45	3
R34-3212	R34-3212	ERS044017	ERR069711	CHBJ02000001	CHBJ02000159	15B/C		15C	199	199	199	8	640200	159	68046	1984	45	3
ND6053	8PY5X	ERS070098	ERR129207	COFO02000001	COFO02000165	15F		15C	199	199	199	8	656525	165	85258	2016	44	3
ND6116	M6AJV	ERS070111	ERR124220	CNYI02000001	CNYI02000172	15F		15C	199	199	199	8	876332	172	58608	2011	38	3
NP7022	WWC1T	ERS070142	ERR124251	CFIU02000001	CFIU02000172	15F		15C	199	199	199	8	936218	172	51800	2010	44	3
R34-3063	R34-3063	ERS043883	ERR065319	CNOK02000001	CNOK02000182	15F		15B	199	199	199	8	486883	182	68971	2057	46	3
57144	057144	ERS044051	ERR069745	CNSK02000001	CNSK02000195	19A		19A	199	199	199	8	750356	195	64801	2078	46	3
80104	080104	ERS044059	ERR069753	CNTC02000001	CNTC02000175	19A		19A	199	199	199	8	836317	175	68967	1968	47	3
135771	135771	ERS044077	ERR069771	CNTN02000001	CNTN02000165	19A		19A	199	199	199	8	768304	165	69133	1967	47	3
148056	148056	ERS044082	ERR069776	CNTZ02000001	CNTZ02000166	19A		19A	1936	1936	1936	8	857827	166	69132	2032	46	0
160449	160449	ERS044087	ERR069781	CNUG02000001	CNUG02000149	19A		19A	199	199	199	8	668543	149	69133	1964	47	3
210162	210162	ERS044100	ERR069794	CNUD02000001	CNUD02000187	19A		19A	1936	1936	1936	8	653397	187	68508	2021	39	3
218186	218186	ERS044102	ERR069796	CNUH02000001	CNUH02000160	19A		19A	199	199	199	8	727078	160	60163	1960	47	3
266035	266035	ERS044111	ERR069805	CIDV02000001	CIDV02000165	19A		19A	199	199	199	8	641832	165	68508	2019	48	3
312034	312034	ERS044123	ERR069817	CNUS02000001	CNUS02000152	19F	19A	19A	199	199	199	8	589224	152	68927	2021	47	3
366293	366293	ERS044137	ERR069831	CNVH02000001	CNVH02000146	19A		19A	199	199	199	8	772939	146	68983	1965	46	3
BR1000	6EO69	ERS069916	ERR129025	COAK02000001	COAK02000239	19A		19A	1756	1756	1756	8	418952	239	39414	2014	36	3
BR1105	RZPNF	ERS069950	ERR129059	COBC02000001	COBC02000146	19A		19A	199	199	199	8	518047	146	58735	1970	46	3
BR1112	6UUPJ	ERS069954	ERR129063	CHXL02000001	CHXL02000154	19A		19A	199	199	199	8	479533	154	60175	1963	44	3
CH2007	BHCQB	ERS069963	ERR129072	COBL02000001	COBL02000121	19A		19A	199	199	199	8	614992	121	68308	1963	47	3
GL3029	RCBGR	ERS069999	ERR129108	CFKC02000001	CFKC02000173	19A		19A	199	199	199	8	555058	173	68189	2048	45	3
GL3036	WNYEL	ERS070003	ERR129112	COCW02000001	COCW02000152	19A		19A	199	199	199	8	494197	152	69143	1967	45	3
GL3060	WLTN8	ERS070009	ERR129118	CODA02000001	CODA02000164	19A		19A	2344	2344	2344	8	639781	164	61840	1979	45	3
GL3066	RL36E	ERS070011	ERR129120	CODE02000001	CODE02000196	19A		19A	199	199	199	8	662815	196	60175	2114	48	2
GL3077	REQGJ	ERS070014	ERR129123	CODU02000001	CODU02000166	19A		19A	10013	199	8563	8	543895	166	69029	1969	39	2
LE4055	LOOXY	ERS070031	ERR129140	CFIW02000001	CFIW02000168	19A		19A	199	199	199	8	558101	168	56962	2020	47	3
LE4059	FUOYQ	ERS070033	ERR129142	CODK02000001	CODK02000195	19A		19A	199	199	199	8	461991	195	60175	2054	42	3
LE4106	Z60YP	ERS070044	ERR129153	CODS02000001	CODS02000150	19A		19A	199	199	199	8	521607	150	69236	1969	47	3
LE4142	DPGZI	ERS070051	ERR129160	COED02000001	COED02000149	19A		19A	199	199	199	8	475325	149	69186	2030	47	3
MD5033	YQUAQ	ERS070061	ERR129170	CFIF02000001	CFIF02000135	19A		19A	199	199	199	8	465051	135	73638	1962	45	3
MD5104	IZQWI	ERS070074	ERR129183	COEW02000001	COEW02000144	19A		19A	667	667	667	8	415931	144	68975	1962	44	3
ND6022	1V4ME	ERS070088	ERR129197	COFJ02000001	COFJ02000198	19A		19A	199	199	199	8	579270	198	46314	1969	38	3
ND6064	VQC7K	ERS070100	ERR129209	CHXI02000001	CHXI02000144	19A		19A	199	199	199	8	582921	144	66146	1970	45	3
ND6139	KMMCT	ERS070125	ERR124234	CNXW02000001	CNXW02000168	19A		19A	199	199	199	8	877923	168	50251	1954	38	3
NP7100	6JSY8	ERS070160	ERR124269	CNZC02000001	CNZC02000221	19A		19A	3279	199	199	8	772668	221	41089	1951	41	3
NP7101	0OH1I	ERS070161	ERR124270	CNZB02000001	CNZB02000184	19A		19A	876	876	876	8	799999	184	69191	2046	42	3
PT8004	LIW28	ERS070171	ERR124280	CNZG02000001	CNZG02000171	19A		19A	667	667	667	8	905301	171	59025	2013	40	3
PT8009	6FXSQ	ERS070172	ERR124281	CNZH02000001	CNZH02000180	19A		19A	667	667	667	8	1063222	180	52132	1956	41	3
PT8014	2EAKR	ERS070175	ERR124284	CNZK02000001	CNZK02000172	19A		19A	199	199	199	8	930530	172	56961	1962	36	3
PT8052	RMEOX	ERS070186	ERR124295	CFLR02000001	CFLR02000173	19A		19A	1673	1673	1673	8	887694	173	50251	1966	41	3
PT8106	ATBLM	ERS070206	ERR124315	CFIE02000001	CFIE02000159	19A		19A	199	199	199	8	455535	159	49130	1985	45	3
R34-3049	R34-3049	ERS043869	ERR065305	CNNU02000001	CNNU02000159	19A		19A	667	667	667	8	671850	159	68918	1966	45	3
R34-3061	R34-3061	ERS043881	ERR065317	CFQR02000001	CFQR02000182	19A		19A	667	667	667	8	499173	182	57189	1947	45	3
R34-3068	R34-3068	ERS043887	ERR065323	CNON02000001	CNON02000156	19A		19A	199	199	199	8	544702	156	69027	1971	47	3
R34-3073	R34-3073	ERS043892	ERR065328	CNOP02000001	CNOP02000173	19A		19A	199	199	199	8	456111	173	69027	2027	43	3
R34-3079	R34-3079	ERS043898	ERR067984	CNWD02000001	CNWD02000161	19A		19A	199	199	199	8	738681	161	68981	1968	47	3
R34-3103	R34-3103	ERS043922	ERR068008	CNWS02000001	CNWS02000207	19A		19A	199	199	199	8	741583	207	56953	2074	45	3
R34-3110	R34-3110	ERS043925	ERR068011	CHWW02000001	CHWW02000157	19A		19A	1935	1935	1935	8	531414	157	68973	1949	46	3
R34-3116	R34-3116	ERS043931	ERR068017	CIIL02000001	CIIL02000166	19A		19A	199	199	199	8	731041	166	69027	1972	46	3
R34-3124	R34-3124	ERS043938	ERR068024	CNWM02000001	CNWM02000171	19A		19A	199	199	199	8	646107	171	60163	1957	47	3
R34-3185	R34-3185	ERS043990	ERR069684	CNQV02000001	CNQV02000177	19A		19A	199	199	199	8	791227	177	69027	1957	44	3
R34-3197	R34-3197	ERS044002	ERR069696	CNRD02000001	CNRD02000164	19A		19A	199	199	199	8	735713	164	71041	1973	46	3
R34-3218	R34-3218	ERS044023	ERR069717	CIDX02000001	CIDX02000166	19A		19A	199	199	199	8	929092	166	69062	1962	47	2
95344	095344	ERS044064	ERR069758	CHXD02000001	CHXD02000176	7		7C		199	199	8	791422	176	64700	1971	33	3
276430	276430	ERS044113	ERR069807	CNUF02000001	CNUF02000133	18C		18C		SLV42	6712	9	555035	133	86170	2009	45	3
154389	154389	ERS044086	ERR069780	CIIJ02000001	CIIJ02000130	23A		23A	436	436	436	9	719341	130	74001	2038	45	3
354557	354557	ERS044132	ERR069826	CNVQ02000001	CNVQ02000141	23A		23A	436	436	436	9	721943	141	84476	2032	45	3
BR1090	ON38S	ERS069946	ERR129055	CHBW02000001	CHBW02000122	23A		23A	42	42	42	9	547753	122	80068	2005	44	3
BR1114	5ZS1X	ERS069956	ERR129065	COBI02000001	COBI02000125	23A		23A	3287	439	439	9	529650	125	84607	2024	44	3
CH2026	9T42W	ERS069967	ERR129076	COBU02000001	COBU02000163	23A		23A	438	438	438	9	563573	163	79466	1981	40	3
CH2061	TEF7S	ERS069973	ERR129082	COBX02000001	COBX02000126	23A		23A	190	190	190	9	602853	126	79182	2013	43	3
CH2116	2AHBE	ERS069989	ERR129098	COCN02000001	COCN02000165	23A		23A	438	438	438	9	547181	165	72647	1995	40	3
GL3023	J9GMM	ERS069997	ERR129106	CFRC02000001	CFRC02000134	23A		23A	1839	1839	1839	9	628593	134	74397	2048	45	3
GL3049	YHPD0	ERS070007	ERR129116	CFLU02000001	CFLU02000113	23A		23A	3285	1839	1839	9	566727	113	80565	2003	46	3
LE4020	CJISL	ERS070024	ERR129133	CODI02000001	CODI02000132	23A		23A	42	42	42	9	483668	132	74087	2008	43	3
LE4058	LIT4Q	ERS070032	ERR129141	CODP02000001	CODP02000123	23A		23A	1839	1839	1839	9	513277	123	84488	2050	46	3
LE4081	ZXPKH	ERS070037	ERR129146	CODO02000001	CODO02000122	23F		23A	42	42	42	9	545528	122	89884	2006	45	3
ND6051	94ORH	ERS070096	ERR129205	COFL02000001	COFL02000120	23A		23A	42	42	42	9	717150	120	95546	2012	45	3
PT8021	Y0160	ERS070178	ERR124287	CNZN02000001	CNZN02000343	23A		23A	3289	6712	6712	9	1057321	343	18594	1978	26	3
R34-3050	R34-3050	ERS043870	ERR065306	CNNW02000001	CNNW02000134	23A		23A	1949	6712	6712	9	660213	134	89286	2010	46	3
R34-3051	R34-3051	ERS043871	ERR065307	CNNX02000001	CNNX02000132	23A		23A	438	438	438	9	593281	132	90651	2007	45	3
R34-3055	R34-3055	ERS043875	ERR065311	CNOB02000001	CNOB02000116	23A		23A	1839	1839	1839	9	465525	116	97954	2011	47	3
R34-3057	R34-3057	ERS043877	ERR065313	CNOC02000001	CNOC02000132	23A		23A	438	438	438	9	482946	132	79454	1997	46	3
R34-3067	R34-3067	ERS043886	ERR065322	CNOM02000001	CNOM02000120	23A		23A	1839	1839	1839	9	549576	120	97720	2002	47	3
R34-3127	R34-3127	ERS043941	ERR068027	CNWX02000001	CNWX02000135	23A		23A	1839	1839	1839	9	481401	135	84477	1998	43	3
R34-3174	R34-3174	ERS043979	ERR067973	CNQG02000001	CNQG02000122	23A		23A	1839	1839	1839	9	661844	122	86185	1996	47	3
R34-3177	R34-3177	ERS043982	ERR067976	CNQI02000001	CNQI02000133	23A		23A	42	42	42	9	611198	133	81077	2011	47	3
248734	248734	ERS044108	ERR069802	CNUZ02000001	CNUZ02000132	23B		23B	439	439	439	9	626347	132	73552	1989	45	3
BR1014	FOR95	ERS069920	ERR129029	COBO02000001	COBO02000110	23B		23B	36	36	36	9	525350	110	97610	2016	42	3
BR1084	QRPPW	ERS069943	ERR129052	COAY02000001	COAY02000102	23B		23B	36	36	36	9	606062	102	80281	2014	44	3
CH2124	C3W41	ERS069991	ERR129100	COCM02000001	COCM02000195	23B		23B	1847	1847	1847	9	535613	195	58297	2089	41	3
GL3024	DYJGL	ERS069998	ERR129107	CFJZ02000001	CFJZ02000126	23B		23B	439	439	439	9	514306	126	73569	2009	44	3
GL3075	WCULS	ERS070013	ERR129122	CFLP02000001	CFLP02000149	23B		23B	439	439	439	9	602400	149	63696	2052	45	3
LE4083	3MB4Z	ERS070038	ERR129147	CHXF02000001	CHXF02000126	23B		23B	439	439	439	9	473096	126	81334	2034	43	3
LE4117	WYMQI	ERS070046	ERR129155	CODY02000001	CODY02000154	23B		23B	439	439	439	9	511369	154	58632	2039	39	3
LE4150	BDGWM	ERS070052	ERR129161	COEE02000001	COEE02000145	23B		23B	1847	1847	1847	9	435042	145	73566	2094	45	3
MD5035	8A4VR	ERS070062	ERR129171	COEN02000001	COEN02000136	23B		23B	36	36	36	9	515661	136	84483	2071	45	3
ND6008	DP07V	ERS070083	ERR129192	COFB02000001	COFB02000137	23B		23B	3281	1448	1448	9	581471	137	74061	2041	34	3
ND6059	7LLCL	ERS070099	ERR129208	CFRL02000001	CFRL02000152	23B		23B	1847	1847	1847	9	628205	152	58264	2099	44	3
ND6115	T7IZ1	ERS070110	ERR124219	CHIR02000001	CHIR02000127	23B		23B	36	36	36	9	981592	127	74457	2018	44	3
ND6144	LFB15	ERS070129	ERR124238	CFKB02000001	CFKB02000139	23B		23B	439	439	439	9	1203429	139	70438	2009	44	3
NP7006	04WB3	ERS070136	ERR124245	CNYE02000001	CNYE02000256	23B		23B	1847	1847	1847	9	1055259	256	48628	2087	42	3
R34-3019	R34-3019	ERS043840	ERR065345	CNPB02000001	CNPB02000176	23B		23B	439	439	439	9	532656	176	63728	2042	47	3
R34-3020	R34-3020	ERS043841	ERR065346	CNOS02000001	CNOS02000156	23B		23B	436	439	439	9	528955	156	65013	2045	46	3
R34-3022	R34-3022	ERS043843	ERR065348	CNOU02000001	CNOU02000171	23B		23B	1847	1847	1847	9	467280	171	59893	2091	42	3
R34-3114	R34-3114	ERS043929	ERR068015	CIKP02000001	CIKP02000140	23B		23B	439	439	439	9	499449	140	58852	2025	46	3
R34-3169	R34-3169	ERS043973	ERR067967	CHBH02000001	CHBH02000154	23B		23B	439	439	439	9	634450	154	57848	2015	45	3
R34-3195	R34-3195	ERS044000	ERR069694	CNRC02000001	CNRC02000153	23B		23B	439	439	439	9	800201	153	77317	2059	45	3
25310	025310	ERS044042	ERR069736	CNTE02000001	CNTE02000118	23F		23F	36	36	36	9	772735	118	94139	2034	45	3
49470	049470	ERS044049	ERR069743	CNSG02000001	CNSG02000138	23F		23F	629	629	629	9	741806	138	86199	1976	45	3
151684	151684	ERS044083	ERR069777	CNUI02000001	CNUI02000134	23F		23F	33	33	33	9	768253	134	113656	2003	41	3
203692	203692	ERS044096	ERR069790	CNTX02000001	CNTX02000130	23F		23F	36	36	36	9	723661	130	97257	2025	43	3
239434	239434	ERS044106	ERR069800	CFLL02000001	CFLL02000125	23F		23F	36	36	36	9	701687	125	95258	2032	42	3
365502	365502	ERS044136	ERR069830	CHBM02000001	CHBM02000160	23F		23F	439	439	439	9	625033	160	104195	2108	43	3
427937	427937	ERS044152	ERR065955	CNPX02000001	CNPX02000151	23F		23F	627	627	627	9	1410541	151	62540	2037	46	3
462956	462956	ERS044163	ERR065966	CNPO02000001	CNPO02000210	23F		23F	36	36	36	9	1471009	210	74439	2019	41	3
R34-3013	R34-3013	ERS043834	ERR065339	CNOW02000001	CNOW02000132	23F		23F	1908	1908	1908	9	484809	132	118278	2045	46	3
R34-3029	R34-3029	ERS043850	ERR065355	CNOZ02000001	CNOZ02000109	23F		23F	36	36	36	9	741213	109	118419	2016	45	3
R34-3076	R34-3076	ERS043895	ERR065331	CNOQ02000001	CNOQ02000102	23F		23F	36	36	36	9	546962	102	122873	1977	46	3
R34-3181	R34-3181	ERS043986	ERR067980	CNQP02000001	CNQP02000144	23F		23F	1909	1909	1909	9	583435	144	122802	1976	43	3
93209	093209	ERS044063	ERR069757	CNTL02000001	CNTL02000120	6C		6C	1379	1379	1379	10	764238	120	74728	1992	45	3
119571	119571	ERS044072	ERR069766	CNTA02000001	CNTA02000137	6C		6C	1390	1390	1390	10	778044	137	79162	1994	45	3
291880	291880	ERS044117	ERR069811	CNUO02000001	CNUO02000165	6C		6C	1390	1390	1390	10	629035	165	79160	2002	45	3
BR1045	NB8E4	ERS069930	ERR129039	COAN02000001	COAN02000156	6C		6C	1390	1390	1390	10	523591	156	65272	1999	43	3
BR1076	QQK9Y	ERS069939	ERR129048	CRYL02000001	CRYL02000106	6C		6C	1379	1379	1379	10	551256	106	123360	1987	45	3
GL3032	SEOTT	ERS070002	ERR129111	COCV02000001	COCV02000132	6C		6C	1379	1379	1379	10	633772	132	71318	1979	41	3
LE4079	ODNWT	ERS070036	ERR129145	CFLY02000001	CFLY02000125	6C		6C	1379	1379	1379	10	630498	125	78375	2001	47	3
LE4100	V5VF1	ERS070043	ERR129152	CODV02000001	CODV02000116	6C		6C	1390	1390	1390	10	435645	116	88368	1999	45	3
MD5031	PHY03	ERS070060	ERR129169	COEL02000001	COEL02000167	6C		6C	1390	1390	1390	10	457980	167	71747	1992	34	3
ND6046	8CRCE	ERS070094	ERR129203	COFK02000001	COFK02000146	6C		6C	1390	1390	1390	10	572506	146	65224	1994	45	3
ND6110	PWGX0	ERS070108	ERR124217	CNXT02000001	CNXT02000165	6C		6C	1379	1379	1379	10	1478923	165	62946	1984	40	3
ND6135	6PU39	ERS070123	ERR124232	CNXZ02000001	CNXZ02000170	6C		6C	1390	1390	1390	10	925659	170	51208	1989	38	3
NP7028	NKLJL	ERS070144	ERR124253	CNYP02000001	CNYP02000211	6C		6C	1379	1379	1379	10	1202456	211	68216	2040	42	3
NP7029	3JK7Y	ERS070145	ERR124254	CNYT02000001	CNYT02000174	6C		6C	1390	1390	1390	10	759341	174	54537	2041	37	3
NP7070	057G3	ERS070155	ERR124264	CNYU02000001	CNYU02000162	6C		6C	1390	1390	1390	10	1435872	162	48360	1997	41	3
NP7078	4XILA	ERS070157	ERR124266	CNZA02000001	CNZA02000167	6C		6C	1379	1379	1379	10	734159	167	71318	1982	38	3
PT8081	LY288	ERS070196	ERR124305	CIIT02000001	CIIT02000152	6C		6C	1379	1379	1379	10	1309262	152	74740	1981	44	2
PT8092	QU2WM	ERS070201	ERR124310	CHXC02000001	CHXC02000156	6C		6C	1379	1379	1379	10	1252811	156	69492	1990	38	3
PT8114	QD0ZV	ERS070208	ERR124317	CHXJ02000001	CHXJ02000141	6C		6C	1390	1390	1390	10	501117	141	71207	1998	45	3
R34-3041	R34-3041	ERS043862	ERR065298	CNNM02000001	CNNM02000151	6C		6C	1390	1390	1390	10	1020710	151	71195	2007	45	3
R34-3060	R34-3060	ERS043880	ERR065316	CNOF02000001	CNOF02000144	6C		6C	1379	1379	1379	10	464158	144	74728	2046	45	3
R34-3072	R34-3072	ERS043891	ERR065327	CNOO02000001	CNOO02000138	6C		6C	1390	1390	1390	10	536053	138	68749	1993	45	3
R34-3089	R34-3089	ERS043908	ERR067994	CNVY02000001	CNVY02000202	6C		6C	1390	1390	1390	10	662413	202	66654	1986	41	3
R34-3092	R34-3092	ERS043911	ERR067997	CNWB02000001	CNWB02000152	Pool I	6C	6C	1390	1390	1390	10	695410	152	68969	2000	45	3
R34-3125	R34-3125	ERS043939	ERR068025	CNWP02000001	CNWP02000180	6C		6C	1390	1390	1390	10	641249	180	47929	2000	41	3
R34-3053	R34-3053	ERS043873	ERR065309	CNNZ02000001	CNNZ02000133	NT		6C	1379	1379	1379	10	581258	133	79177	2051	45	3
7649	007649	ERS044039	ERR069733	CNSZ02000001	CNSZ02000102	35B		35B	558	558	558	11	645576	102	80308	1928	45	3
67094	067094	ERS044055	ERR069749	CNSL02000001	CNSL02000116	35B		35B	1946	558	558	11	674433	116	68288	1903	44	3
379678	379678	ERS044140	ERR069834	CNUX02000001	CNUX02000158	35B		35B	558	558	558	11	618903	158	47273	1967	41	3
BR1029	T8Z8O	ERS069926	ERR129035	COAL02000001	COAL02000109	35B		35B	558	558	558	11	480655	109	68301	1914	44	3
CH2016	F77ZH	ERS069965	ERR129074	CHXB02000001	CHXB02000118	35B		35B	558	558	558	11	516125	118	80523	1968	42	3
CH2074	4K4C9	ERS069977	ERR129086	COCI02000001	COCI02000152	35B		35B	558	558	558	11	575562	152	53336	1921	40	3
CH2075	QWSZT	ERS069978	ERR129087	COCD02000001	COCD02000108	35B		35B	558	558	558	11	524683	108	71794	1917	42	3
CH2083	4PYM0	ERS069980	ERR129089	COCG02000001	COCG02000169	35B		35B	558	558	558	11	621661	169	39912	1917	41	3
CH2123	WAMFH	ERS069990	ERR129099	COCQ02000001	COCQ02000117	35B		35B	558	558	558	11	552266	117	64020	1918	44	3
GL3019	W8IHX	ERS069995	ERR129104	COCS02000001	COCS02000166	35B		35B	558	558	558	11	600023	166	46254	1915	38	3
GL3061	9D25H	ERS070010	ERR129119	CODF02000001	CODF02000144	35B		35B	558	558	558	11	642988	144	52254	1914	40	3
LE4021	P1NM2	ERS070025	ERR129134	CODJ02000001	CODJ02000133	35B		35B	3277	3277	3277	11	535581	133	53334	1925	40	3
LE4062	0U64I	ERS070034	ERR129143	CODM02000001	CODM02000124	35B		35B	558	558	558	11	560663	124	67445	1916	40	3
LE4084	UTEDZ	ERS070039	ERR129148	CODQ02000001	CODQ02000190	35B		35B	558	558	558	11	482101	190	43583	1912	30	3
MD5008	B1KMB	ERS070054	ERR129163	COEK02000001	COEK02000161	35B		35B	558	558	558	11	557353	161	51757	1936	40	3
MD5042	AFVC5	ERS070064	ERR129173	COEP02000001	COEP02000138	35B		35B	558	558	558	11	513828	138	56186	1904	38	3
MD5081	REAOU	ERS070070	ERR129179	CHXN02000001	CHXN02000128	35B		35B	558	558	558	11	525989	128	67445	1912	40	3
ND6038	0NWX9	ERS070091	ERR129200	COFI02000001	COFI02000147	35B		35B	558	558	558	11	536535	147	49320	1921	41	3
ND6141	1VDX8	ERS070127	ERR124236	CNYG02000001	CNYG02000171	35B		35B	558	558	558	11	890199	171	46882	1912	40	2
ND6153	CCV1H	ERS070132	ERR124241	CNYD02000001	CNYD02000180	35B		35B	558	558	558	11	1356930	180	40436	1910	40	3
NP7020	W9GKO	ERS070141	ERR124250	CNYR02000001	CNYR02000141	35B		35B	558	558	558	11	986715	141	67171	1988	41	0
NP7109	LS3OB	ERS070164	ERR124273	CFJU02000001	CFJU02000211	35B		35B	558	558	558	11	1482671	211	39912	1912	40	2
NP7113	RS9D2	ERS070166	ERR124275	CNZF02000001	CNZF02000316	35B		35B	558	558	558	11	1675064	316	21028	1901	31	3
NP7122	6893Z	ERS070168	ERR124277	CFQY02000001	CFQY02000193	35B		35B	558	558	558	11	845238	193	39914	1911	30	3
PT8003	81LMX	ERS070170	ERR124279	CNZI02000001	CNZI02000208	35B		35B	558	558	558	11	786840	208	40209	1910	39	3
PT8035	BZ2I7	ERS070181	ERR124290	CNZO02000001	CNZO02000220	35B		35B	558	558	558	11	1119263	220	34345	1905	34	3
PT8040	O0RHB	ERS070184	ERR124293	CHBS02000001	CHBS02000240	35B		35B	558	558	558	11	857518	240	32540	1920	30	3
PT8064	O61U7	ERS070190	ERR124299	CNZT02000001	CNZT02000253	35B		35B	558	558	558	11	1020004	253	31937	1910	29	3
PT8069	O8I1E	ERS070192	ERR124301	CNZV02000001	CNZV02000240	35B		35B	558	558	558	11	1139763	240	30607	1906	32	3
PT8107	JBYFY	ERS070207	ERR124316	COAG02000001	COAG02000101	35B		35B	558	558	558	11	390709	101	67446	1917	44	3
R34-3025	R34-3025	ERS043846	ERR065351	CNPL02000001	CNPL02000147	35B		35B	558	558	558	11	496882	147	49192	1914	43	3
R34-3044	R34-3044	ERS043864	ERR065300	CNNL02000001	CNNL02000120	35B		35B	558	558	558	11	552380	120	68288	1922	45	3
R34-3069	R34-3069	ERS043888	ERR065324	CNOL02000001	CNOL02000127	35B		35B	558	558	558	11	566136	127	68090	1930	41	3
R34-3074	R34-3074	ERS043893	ERR065329	CNOV02000001	CNOV02000167	NT	35B	35B	558	558	558	11	473228	167	43706	1920	38	3
R34-3083	R34-3083	ERS043902	ERR067988	CNWN02000001	CNWN02000132	35B		35B	558	558	558	11	671702	132	67433	1924	41	3
R34-3087	R34-3087	ERS043906	ERR067992	CNVX02000001	CNVX02000118	35B		35B	558	558	558	11	582794	118	68289	1920	41	3
R34-3097	R34-3097	ERS043916	ERR068002	CNWG02000001	CNWG02000226	35B		35B	558	558	558	11	659561	226	38025	1912	30	3
R34-3119	R34-3119	ERS043933	ERR068019	CNWT02000001	CNWT02000111	35B		35B	558	558	558	11	598720	111	80487	1962	45	3
R34-3131	R34-3131	ERS043943	ERR068029	CNXA02000001	CNXA02000127	35B		35B	558	558	558	11	683965	127	70434	1962	45	3
R34-3139	R34-3139	ERS043947	ERR068033	CNXR02000001	CNXR02000163	35B		35B	558	558	558	11	622738	163	45107	1914	41	3
R34-3164	R34-3164	ERS043968	ERR067962	CNQO02000001	CNQO02000143	35B	Pool G	35B	1947	1947	1947	11	606548	143	51654	1922	40	3
R34-3165	R34-3165	ERS043969	ERR067963	CNQN02000001	CNQN02000116	35B		35B	558	558	558	11	581140	116	68288	1976	45	3
R34-3172	R34-3172	ERS043976	ERR067970	CNQC02000001	CNQC02000182	35B		35B	1947	3632	3632	11	547613	182	41812	1925	41	3
R34-3204	R34-3204	ERS044009	ERR069703	CNQS02000001	CNQS02000159	35B		35B	558	558	558	11	738391	159	67700	2012	41	3
R34-3227	R34-3227	ERS044032	ERR069726	CNRW02000001	CNRW02000170	35B		35B	558	558	558	11	777195	170	47314	1931	42	3
R34-3229	R34-3229	ERS044034	ERR069728	CNRY02000001	CNRY02000138	35B		35B	558	558	558	11	807583	138	56108	1914	42	3
255210	255210	ERS044109	ERR069803	CNVE02000001	CNVE02000206		NT	NT		449	449	12	521097	206	39906	2097	45	3
403790	403790	ERS044147	ERR069841	CNVO02000001	CNVO02000197	NT		NT		1054	7803	12	621882	197	40790	2040	45	3
462746	462746	ERS044162	ERR065965	CNPK02000001	CNPK02000180	NT		NT	448	448	448	12	1434835	180	54927	2084	45	3
508907	508907	ERS044171	ERR065974	CNPU02000001	CNPU02000205	NT		NT	448	448	448	12	1479387	205	54929	2081	45	3
LE4012	68G2D	ERS070021	ERR129130	CODC02000001	CODC02000201	NT		NT	62	448	448	12	765506	201	53870	2087	34	3
PT8000	WVCE6	ERS070169	ERR124278	CFQQ02000001	CFQQ02000209	NT		NT	3278	448	448	12	1107892	209	49980	2081	44	3
R34-3015	R34-3015	ERS043836	ERR065341	CNPF02000001	CNPF02000171	NT		NT	448	448	448	12	554434	171	50610	2077	45	3
R34-3032	R34-3032	ERS043853	ERR065289	CFQL02000001	CFQL02000166	Pool I	NT	NT		448	448	12	664900	166	53916	2089	45	3
R34-3039	R34-3039	ERS043860	ERR065296	CNNO02000001	CNNO02000165	6B	NT	NT	448	448	448	12	646546	165	54902	2086	45	3
R34-3088	R34-3088	ERS043907	ERR067993	CFJY02000001	CFJY02000211		NT	NT		344	344	12	814267	211	40190	2089	45	3
15445	015445	ERS044040	ERR069734	CNTB02000001	CNTB02000210	6A		6B	1876	1876	1876	13	710738	210	50116	1983	42	3
42861	042861	ERS044047	ERR069741	CNSE02000001	CNSE02000269	6A		6B	1911	10036	10036	13	685819	269	39880	1983	32	3
101058	101058	ERS044065	ERR069759	CHBL02000001	CHBL02000162	6A		6B	690	690	690	13	882416	162	59787	1989	52	3
166637	166637	ERS044090	ERR069784	CNTU02000001	CNTU02000140		6	6B		473	473	13	658118	140	63964	2000	46	3
181272	181272	ERS044094	ERR069788	CNTW02000001	CNTW02000159	6A		6B	1876	1876	1876	13	701590	159	59255	1994	46	3
359370	359370	ERS044133	ERR069827	CNVT02000001	CNVT02000171	6A		6B	1926	1876	1876	13	740093	171	57397	1991	46	3
420881	420881	ERS044150	ERR065953	CIDS02000001	CIDS02000271	6A		6B	1940	1940	1940	13	1327340	271	39783	1986	31	3
CH2090	0TN5H	ERS069983	ERR129092	COCJ02000001	COCJ02000186	6A		6B	1876	1876	1876	13	566419	186	53289	2035	44	3
R34-3027	R34-3027	ERS043848	ERR065353	CNPD02000001	CNPD02000178	6A		6B	473	473	473	13	563530	178	54094	1987	42	3
R34-3096	R34-3096	ERS043915	ERR068001	CNWF02000001	CNWF02000192	6A		6B	1928	1928	1928	13	643363	192	56978	1976	42	3
R34-3099	R34-3099	ERS043918	ERR068004	CFIP02000001	CFIP02000154	6A		6B	1927	1927	1927	13	714180	154	65660	2058	46	3
R34-3151	R34-3151	ERS043957	ERR068043	CNXG02000001	CNXG02000253	6A		6B	1917	1876	1876	13	622561	253	46473	1982	44	3
R34-3180	R34-3180	ERS043985	ERR067979	CNQL02000001	CNQL02000260	6A		6B	690	690	690	13	554946	260	40320	1977	40	3
R34-3210	R34-3210	ERS044015	ERR069709	CHIO02000001	CHIO02000157	6A		6B	1876	1876	1876	13	752877	157	63730	2001	46	3
R34-3211	R34-3211	ERS044016	ERR069710	CNSA02000001	CNSA02000196	6A		6B	1876	1876	1876	13	664068	196	54094	1983	39	3
R34-3100	R34-3100	ERS043919	ERR068005	CHWV02000001	CHWV02000159	6B		6B	690	690	690	13	621229	159	59787	1996	52	3
BR1008	CM917	ERS069918	ERR129027	COBB02000001	COBB02000217	6C		6C	473	473	473	13	608916	217	48780	1994	40	2
GL3043	FU8OL	ERS070006	ERR129115	COCZ02000001	COCZ02000216	6C		6C	473	473	473	13	488273	216	48037	2049	42	3
ND6119	5W908	ERS070114	ERR124223	CNYX02000001	CNYX02000180	6C		6C	473	473	473	13	898992	180	50284	1990	45	3
51715	051715	ERS044050	ERR069744	CNSO02000001	CNSO02000158	6A		6A	376	376	376	14	842144	158	54645	2041	42	3
65435	065435	ERS044053	ERR069747	CNSN02000001	CNSN02000162	6A		6A	376	376	376	14	715450	162	58082	2004	42	3
108480	108480	ERS044069	ERR069763	CNSR02000001	CNSR02000161	6A		6A	376	376	376	14	907154	161	57928	2014	42	3
179743	179743	ERS044093	ERR069787	CNTV02000001	CNTV02000163	6A		6A	376	376	376	14	714250	163	55893	2006	42	3
342672	342672	ERS044131	ERR069825	CNVS02000001	CNVS02000175	6A		6A	1922	1922	1922	14	584422	175	46950	1992	42	3
448643	448643	ERS044160	ERR065963	CNPN02000001	CNPN02000186	6A		6A	376	376	376	14	1238469	186	40684	2038	38	3
LE4007	CXQ2T	ERS070019	ERR129128	COEM02000001	COEM02000189	6A		6B	1538	1538	1538	14	538853	189	40812	2047	38	3
PT8074	AI3GZ	ERS070194	ERR124303	CHBU02000001	CHBU02000177	6A		6A	376	376	376	14	865534	177	54657	2033	43	3
PT8105	2RLC4	ERS070205	ERR124314	COAE02000001	COAE02000167	6A		6B	376	376	376	14	545326	167	54654	2044	35	3
R34-3168	R34-3168	ERS043972	ERR067966	CNQT02000001	CNQT02000231	6A		6A	376	376	376	14	544245	231	40800	2029	37	3
R34-3171	R34-3171	ERS043975	ERR067969	CNQE02000001	CNQE02000256	6A		6A	376	376	376	14	521771	256	34437	2024	34	3
R34-3201	R34-3201	ERS044006	ERR069700	CNQW02000001	CNQW02000360	6A		6A	1921	1921	1921	14	808590	360	22244	2045	30	3
BR1022-1	DH8X5-1	ERS069922	ERR129031	COBR02000001	COBR02000251	19A		19A	320	320	320	15	570719	251	33714	1976	36	3
BR1036	06URQ	ERS069927	ERR129036	COAJ02000001	COAJ02000232	19A		19A	320	320	320	15	519594	232	39129	1977	43	3
BR1080	NFPTS	ERS069940	ERR129049	CFQT02000001	CFQT02000156	19A		19A	320	320	320	15	544707	156	59177	1974	47	3
CH2017	1QJAP	ERS069966	ERR129075	COBV02000001	COBV02000248	19A		19A	320	320	320	15	519029	248	35971	1938	34	3
ND6010	9SKOT	ERS070084	ERR129193	COFD02000001	COFD02000160	19A		19A	320	320	320	15	515723	160	55884	1977	42	3
ND6118	OQTRJ	ERS070113	ERR124222	CNYW02000001	CNYW02000166	19A		19A	320	320	320	15	883212	166	48716	1938	43	3
ND6137	LABTO	ERS070124	ERR124233	CNXV02000001	CNXV02000149	19A		19A	320	320	320	15	865637	149	56124	1969	45	3
NP7047	CHP0M	ERS070146	ERR124255	CNYL02000001	CNYL02000337	19A		19A	1451	1451	1451	15	515569	337	21839	1998	31	3
R34-3221	R34-3221	ERS044026	ERR069720	CNRR02000001	CNRR02000170	19A		19A	320	320	320	15	732768	170	59783	1939	42	3
105542	105542	ERS044068	ERR069762	CFQW02000001	CFQW02000161	9A	19F	19F	1945	1945	1945	15	704891	161	57389	1952	46	2
208866	208866	ERS044097	ERR069791	CNUA02000001	CNUA02000163	19F		19F	236	236	236	15	711884	163	62950	1946	46	3
209930	209930	ERS044099	ERR069793	CNUB02000001	CNUB02000179	19F		19F	236	236	236	15	692143	179	48868	1942	44	3
404463	404463	ERS044148	ERR069842	CHBN02000001	CHBN02000173	19F		19F	236	236	236	15	807881	173	56367	1942	46	3
416185	416185	ERS044149	ERR069843	CNVP02000001	CNVP02000227	19F		19F	320	320	320	15	747175	227	56601	1983	41	3
503574	503574	ERS044170	ERR065973	CNPT02000001	CNPT02000162	19F		19F	320	320	320	15	1443326	162	58805	1986	44	3
BR1111	6GU7V	ERS069953	ERR129062	COBF02000001	COBF02000183	19F		19F	3292	3292	3292	15	553921	183	55163	2000	43	3
CH2033	WMK3T	ERS069969	ERR129078	CFRJ02000001	CFRJ02000186	19F		19F	236	236	236	15	531232	186	50718	1973	42	3
R34-3058	R34-3058	ERS043878	ERR065314	CNOD02000001	CNOD02000172	19F		19F	236	236	236	15	444412	172	57390	1992	46	3
R34-3085	R34-3085	ERS043904	ERR067990	CHBQ02000001	CHBQ02000269	19F		19F	271	271	271	15	790622	269	33792	1972	38	3
R34-3094	R34-3094	ERS043913	ERR067999	CHBR02000001	CHBR02000162	19F		19F	236	236	236	15	671606	162	57389	2016	47	3
R34-3098	R34-3098	ERS043917	ERR068003	CNWH02000001	CNWH02000166	19F		19F	1941	1941	1941	15	597150	166	62940	1949	47	3
R34-3141	R34-3141	ERS043949	ERR068035	CNXS02000001	CNXS02000299	19F		19F	1937	271	271	15	662233	299	28410	1974	31	3
R34-3142	R34-3142	ERS043950	ERR068036	CFRK02000001	CFRK02000305	19F		19F	271	271	271	15	684428	305	24262	1969	31	3
R34-3200	R34-3200	ERS044005	ERR069699	CHBG02000001	CHBG02000149	19F		19F	236	236	236	15	779663	149	57389	1959	46	3
R34-3207	R34-3207	ERS044012	ERR069706	CHWU02000001	CHWU02000166	19F		19F	1943	1943	1943	15	804061	166	56376	1989	45	3
65645	065645	ERS044054	ERR069748	CNSP02000001	CNSP02000120	3		3	180	180	180	16	752562	120	90828	2001	46	3
72511	072511	ERS044056	ERR069750	CNSV02000001	CNSV02000124		3	3		180	180	16	897874	124	91041	1953	43	3
369820	369820	ERS044138	ERR069832	CNVG02000001	CNVG02000119	3		3	180	180	180	16	826096	119	95228	1987	39	3
MD5090	P213M	ERS070071	ERR129180	COEV02000001	COEV02000122	3		3	180	180	180	16	483715	122	97568	2003	43	3
ND6097	LDD87	ERS070103	ERR129212	COFU02000001	COFU02000128	3		3	180	180	180	16	1020942	128	91059	1987	42	1
R34-3078	R34-3078	ERS043897	ERR067983	CNVW02000001	CNVW02000124	3		3	180	180	180	16	712646	124	91025	1950	43	3
R34-3084	R34-3084	ERS043903	ERR067989	CFLM02000001	CFLM02000251	3		3	180	180	180	16	762742	251	58581	2046	41	3
R34-3101	R34-3101	ERS043920	ERR068006	CNWU02000001	CNWU02000122	3		3	180	180	180	16	679544	122	91029	1950	43	3
R34-3190	R34-3190	ERS043995	ERR069689	CNRJ02000001	CNRJ02000110	3		3	180	180	180	16	738923	110	96268	1984	44	3
R34-3202	R34-3202	ERS044007	ERR069701	CNRH02000001	CNRH02000134	3		3	180	180	180	16	805360	134	91041	1995	43	3
R34-3217	R34-3217	ERS044022	ERR069716	CNRP02000001	CNRP02000117	3		3	180	180	180	16	650169	117	91029	1956	43	3
142534	142534	ERS044078	ERR069772	CNTO02000001	CNTO02000185	14		14	124	124	124	16	785710	185	38979	1994	46	3
268660	268660	ERS044112	ERR069806	CNVB02000001	CNVB02000174	14		14	124	124	124	16	662547	174	40732	1989	45	3
306435	306435	ERS044122	ERR069816	CNUV02000001	CNUV02000182	14		14	124	124	124	16	578420	182	40732	1997	45	3
341365	341365	ERS044130	ERR069824	CNVR02000001	CNVR02000197	14		14	124	124	124	16	713927	197	43426	2054	46	3
430772	430772	ERS044153	ERR065956	CIIR02000001	CIIR02000188	14		14	124	124	124	16	1217194	188	40138	1998	46	3
R34-3230	R34-3230	ERS044035	ERR069729	CNSD02000001	CNSD02000182	14		14	124	124	124	16	840724	182	40732	2030	45	3
R34-3231	R34-3231	ERS044036	ERR069730	CNSU02000001	CNSU02000184	14		14	124	124	124	16	715524	184	55853	2038	46	3
GL3031	VODW3	ERS070001	ERR129110	COCU02000001	COCU02000138	21		21	432	432	432	16	569706	138	67367	2042	44	3
BR1101	E0ZKK	ERS069948	ERR129057	COBA02000001	COBA02000131	31		31	568	568	568	16	545690	131	52285	1957	44	3
LE4030	N8YA4	ERS070026	ERR129135	CODH02000001	CODH02000119	31		31	568	568	568	16	524480	119	52473	1952	44	3
PT8093	YSOFP	ERS070202	ERR124311	COAC02000001	COAC02000120	31		31	568	568	568	16	1058147	120	52277	1960	45	3
R34-3033	R34-3033	ERS043854	ERR065290	CNNK02000001	CNNK02000130	31		31	568	568	568	16	616222	130	52596	1957	45	3
438180	438180	ERS044156	ERR065959	CNQA02000001	CNQA02000194	34		34	547	547	547	16	1396389	194	59325	2070	41	3
BR1041	NA03L	ERS069929	ERR129038	COAP02000001	COAP02000183	34		34	547	547	547	16	542908	183	73649	2045	40	3
BR1115	DR1PD	ERS069957	ERR129066	COBK02000001	COBK02000126	34		34	547	547	547	16	563613	126	76434	2046	45	3
R34-3048	R34-3048	ERS043868	ERR065304	CNNV02000001	CNNV02000142	34		34	1931	547	547	16	701664	142	73930	2067	45	3
R34-3115	R34-3115	ERS043930	ERR068016	CNWK02000001	CNWK02000142	34		34	547	547	547	16	609572	142	76543	2067	45	3
R34-3176	R34-3176	ERS043981	ERR067975	CNQH02000001	CNQH02000237	34		34	1902	1902	1902	16	663022	237	44714	2096	39	3
304793	304793	ERS044121	ERR069815	CNUY02000001	CNUY02000166	9N	Pool D	37	447	447	6942	16	596507	166	48962	1946	43	3
LE4099	F5UY4	ERS070042	ERR129151	CODT02000001	CODT02000143	37		37	447	447	6942	16	417920	143	49010	1942	45	3
147587	147587	ERS044081	ERR069775	CNTR02000001	CNTR02000264	38		38	393	393	393	16	741838	264	45303	2036	38	3
BR1048	HU4AA	ERS069931	ERR129040	COAO02000001	COAO02000265	38		38	393	393	393	16	534430	265	32187	2068	38	3
BR1098	8PNF1	ERS069947	ERR129056	CFRF02000001	CFRF02000204	38		38	393	393	393	16	552230	204	39157	2023	42	2
GL3078	J29X8	ERS070015	ERR129124	COEC02000001	COEC02000189	NT	38	38	393	393	393	16	533034	189	45340	2047	45	3
ND6005	K8121	ERS070079	ERR129188	COFC02000001	COFC02000197	38		38	393	393	393	16	483648	197	39628	2054	45	3
R34-3046	R34-3046	ERS043866	ERR065302	CNNN02000001	CNNN02000235	38		38	393	393	393	16	587582	235	45392	2047	41	3
R34-3065	R34-3065	ERS043885	ERR065321	CNOJ02000001	CNOJ02000199	25A	38	38	393	393	393	16	531234	199	49022	2051	45	3
R34-3146	R34-3146	ERS043953	ERR068039	CNXC02000001	CNXC02000195	6C	38	38	1390	393	393	16	571351	195	45320	2038	45	3
277394	277394	ERS044114	ERR069808	CNUQ02000001	CNUQ02000206	11A		11D	1934	7661	7661	16	610477	206	57136	2088	42	3
333793	333793	ERS044126	ERR069820	CNUP02000001	CNUP02000141	15A		15A	817	817	817	16	733666	141	56397	1954	45	3
NP7004	18FDW	ERS070135	ERR124244	CNYA02000001	CNYA02000142	15B/C		15C	346	346	346	16	895142	142	63816	2005	43	3
BR1050	X703J	ERS069932	ERR129041	COAQ02000001	COAQ02000169	16F		16F	995	995	995	16	597707	169	65882	2021	43	3
MD5096	2W7ME	ERS070072	ERR129181	COET02000001	COET02000149	16F		16F	570	570	570	16	527284	149	80141	2023	34	3
MD5106	SHTPB	ERS070075	ERR129184	COEX02000001	COEX02000167	16F		16F	659	659	659	16	538101	167	74046	2072	44	3
NP7012	M66VO	ERS070138	ERR124247	CNYJ02000001	CNYJ02000197	16F		16F	1840	1840	1840	16	1444341	197	63248	2086	44	3
NP7103	2Y1CQ	ERS070162	ERR124271	CNZD02000001	CNZD02000133	16F		16F	659	659	659	16	711094	133	73934	1961	44	3
PT8029	RD7KX	ERS070180	ERR124289	CNZM02000001	CNZM02000203	16F		16F	659	659	659	16	914843	203	52928	2061	39	3
PT8090	BA5TC	ERS070200	ERR124309	CNZY02000001	CNZY02000191	16F		16F	659	659	659	16	1161912	191	57166	2078	45	3
R34-3062	R34-3062	ERS043882	ERR065318	CNOH02000001	CNOH02000180	16F		16F	659	659	659	16	471228	180	80496	1976	45	3
ND6111	GWTK7	ERS070109	ERR124218	CHBP02000001	CHBP02000162	17F		17F	392	392	392	16	1436662	162	57903	1963	44	3
NP7026	9LUM5	ERS070143	ERR124252	CFRB02000001	CFRB02000133	17F		17F	2355	2355	8563	16	1437060	133	74364	1954	46	3
NP7057	3XUC4	ERS070149	ERR124258	CNYS02000001	CNYS02000142	17F		17F	392	392	392	16	841522	142	66633	1971	43	3
PT8082	E3GXY	ERS070197	ERR124306	CIEC02000001	CIEC02000145	17F		17F	392	392	392	16	1344486	145	48946	1986	45	3
R34-3056	R34-3056	ERS043876	ERR065312	CNOE02000001	CNOE02000138	17F		17F	1924	1924	1924	16	442380	138	65850	1996	45	3
40241	040241	ERS044045	ERR069739	CNSF02000001	CNSF02000205	18C		18C	1923	1923	1923	16	797963	205	59905	2070	46	3
303656	303656	ERS044120	ERR069814	CHBK02000001	CHBK02000135	18C		18C	1948	496	496	16	652704	135	75300	1997	45	3
R34-3059	R34-3059	ERS043879	ERR065315	CNOG02000001	CNOG02000176	37	18C	18C	1073	1073	1073	16	494629	176	79858	2069	46	3
R34-3102	R34-3102	ERS043921	ERR068007	CNWR02000001	CNWR02000142	18C		18C	496	496	496	16	589985	142	80570	1992	44	3
R34-3196	R34-3196	ERS044001	ERR069695	CHWS02000001	CHWS02000185	18C		18C	113	113	113	16	770603	185	88139	2024	47	3
BR1012	26IET	ERS069919	ERR129028	COBH02000001	COBH02000183	19A		19A	695	SLV695	695	16	549104	183	39572	2072	47	3
BR1019	FRIZ4	ERS069921	ERR129030	COBN02000001	COBN02000184	19A		19A	36	SLV695	695	16	516806	184	39572	2071	46	3
CH2067	X71H5	ERS069974	ERR129083	COBZ02000001	COBZ02000203	19A		19A	695	SLV695	695	16	566926	203	42951	2080	46	3
CH2069	7NA98	ERS069975	ERR129084	COCB02000001	COCB02000143	19A		19A	415	415	415	16	698246	143	56417	1994	44	3
GL3015	Y6BSG	ERS069994	ERR129103	COCR02000001	COCR02000228	19A		19A	695	SLV695	695	16	583440	228	39580	2132	46	3
LE4038	3UTO8	ERS070027	ERR129136	CODN02000001	CODN02000176	19A		19A	695	SLV695	695	16	539693	176	37541	2072	46	3
LE4047	6U8ZJ	ERS070029	ERR129138	CODL02000001	CODL02000201	19A		19A	180	SLV695	695	16	1027130	201	35497	2067	46	2
ND6042	3GWV1	ERS070093	ERR129202	COFP02000001	COFP02000222	19A		19A	695	SLV695	695	16	501324	222	42927	2118	46	3
ND6109	MFII0	ERS070107	ERR129216	COFY02000001	COFY02000174	19A		19A	695	SLV695	695	16	351551	174	42951	2080	46	3
R34-3037	R34-3037	ERS043858	ERR065294	CNNS02000001	CNNS02000203	19A		19A	1944	1944	1944	16	557513	203	48681	2038	41	3
R34-3183	R34-3183	ERS043988	ERR067982	CFQP02000001	CFQP02000130	19A		19A	276	276	10194	16	602182	130	78511	1994	45	3
27999	027999	ERS044043	ERR069737	CNSS02000001	CNSS02000273	19F		19F	1340	1340	1340	16	782294	273	57970	2124	46	3
209464	209464	ERS044098	ERR069792	CNTY02000001	CNTY02000196	19F		19F	425	425	425	16	696536	196	46435	1993	45	3
284968	284968	ERS044116	ERR069810	CNUL02000001	CNUL02000174	19F		19F	425	425	425	16	649536	174	47632	1996	46	3
439699	439699	ERS044157	ERR065960	CFQS02000001	CFQS02000201	19F		19F	654	654	654	16	1403097	201	48219	2008	46	3
BR1065	DALR8	ERS069936	ERR129045	COAT02000001	COAT02000262	19F		19F	426	426	426	16	547973	262	42584	2126	42	3
BR1081	SQADW	ERS069941	ERR129050	COAW02000001	COAW02000178	19F		19F	654	654	654	16	551841	178	46467	2012	47	3
CH2105	QIKYS	ERS069985	ERR129094	COCL02000001	COCL02000190	19F	19F	19F	43	43	43	16	560697	190	44749	2031	45	3
CH2106	Q0413	ERS069986	ERR129095	CXOE01000001	CXOE01000178	NT	19F	19F	43	43	43	16	530890	178	48337	2021	46	3
ND6052	TJRUQ	ERS070097	ERR129206	COFM02000001	COFM02000200	19F		19F	43	43	43	16	629196	200	69065	2035	37	3
R34-3028	R34-3028	ERS043849	ERR065354	CNOX02000001	CNOX02000178	19F		19F	425	425	425	16	606774	178	47404	2050	45	3
R34-3043	R34-3043	ERS043863	ERR065299	CNNR02000001	CNNR02000179	19F		19F	654	654	654	16	556874	179	48619	1964	46	3
R34-3070	R34-3070	ERS043889	ERR065325	CHIN02000001	CHIN02000226	19F		19F	654	654	654	16	506861	226	46277	2053	47	3
R34-3113	R34-3113	ERS043928	ERR068014	CNWZ02000001	CNWZ02000181	19F		19F	43	43	43	16	579564	181	58752	1970	45	3
R34-3137	R34-3137	ERS043945	ERR068031	CFIQ02000001	CFIQ02000190	19F		19F	425	425	425	16	678105	190	51413	2053	46	3
R34-3193	R34-3193	ERS043998	ERR069692	CNQY02000001	CNQY02000181	19F		19F	1903	1903	1903	16	721954	181	50254	2001	45	3
R34-3198	R34-3198	ERS044003	ERR069697	CNQZ02000001	CNQZ02000187	19F		19F	425	425	425	16	656612	187	50412	2061	47	3
R34-3224	R34-3224	ERS044029	ERR069723	CNRV02000001	CNRV02000167	19F		19F	425	425	425	16	783924	167	61628	2011	45	3
44744	044744	ERS044048	ERR069742	CNSJ02000001	CNSJ02000175		23	23F		242	242	16	784856	175	63365	2064	46	3
173015	173015	ERS044091	ERR069785	CNTS02000001	CNTS02000162	23F		23F	81	81	81	16	772262	162	69767	2053	45	3
218229	218229	ERS044103	ERR069797	CNUC02000001	CNUC02000187	23F		23F	81	81	81	16	760163	187	48689	2058	45	3
334847	334847	ERS044127	ERR069821	CNVF02000001	CNVF02000177	23F		23F	81	81	81	16	695521	177	58491	2059	45	3
144370	144370	ERS044079	ERR069773	CNTF02000001	CNTF02000231	25A		38	393	393	393	16	916919	231	36104	2044	35	3
MD5107	OZJ9F	ERS070076	ERR129185	COEZ02000001	COEZ02000100	33A		33F	2705	2705	2705	16	328196	100	122092	2022	44	3
187406	187406	ERS044095	ERR069789	CIEE02000001	CIEE02000122	33		33F	100	100	100	16	591847	122	84375	1992	43	3
NP7053	ZPN7P	ERS070148	ERR124257	CNYQ02000001	CNYQ02000120	33F		33F	2705	2705	2705	16	829937	120	97009	2013	44	3
R34-3121	R34-3121	ERS043935	ERR068021	CNWQ02000001	CNWQ02000125	33F		33F	100	100	100	16	692351	125	74621	1940	45	3
R34-3173	R34-3173	ERS043978	ERR067972	CNQD02000001	CNQD02000128	33F		33F	100	100	100	16	706317	128	75405	1982	45	3
R34-3023	R34-3023	ERS043844	ERR065349	CNPM02000001	CNPM02000184	35B		35B	452	452	452	16	534120	184	50437	1968	45	3
R34-3122	R34-3122	ERS043936	ERR068022	CVKI02000001	CVKI02000195	35B		35B	452	452	452	16	673207	195	54480	1965	45	3
R34-3223	R34-3223	ERS044028	ERR069722	CNRT02000001	CNRT02000180	35B		35B	452	452	452	16	784915	180	58183	1976	45	3
110093	110093	ERS044070	ERR069764	CNSQ02000001	CNSQ02000259	6A		6B	1939	1939	1939	16	805312	259	46223	2074	46	3
R34-3026	R34-3026	ERS043847	ERR065352	CNPC02000001	CNPC02000252	6A		6A	242	242	242	16	578246	252	40403	2030	41	3
18044	018044	ERS044041	ERR069735	CNTD02000001	CNTD02000174	6B		6C	1518	1518	1518	16	837914	174	52440	2077	46	3
160986	160986	ERS044088	ERR069782	CNUM02000001	CNUM02000168	6B		6A	1536	1536	1536	16	748659	168	68783	2007	45	3
302649	302649	ERS044119	ERR069813	CNUW02000001	CNUW02000187	6B		6A	146	146	146	16	671199	187	57742	2059	45	3
323485	323485	ERS044125	ERR069819	CFQU02000001	CFQU02000215	6B		NT	146	146	146	16	627195	215	50634	2047	41	3
388483	388483	ERS044144	ERR069838	CNVK02000001	CNVK02000216	6B		NT	146	146	146	16	789543	216	40936	1998	34	3
397079	397079	ERS044146	ERR069840	CNVN02000001	CNVN02000192	6B		6A	315	315	315	16	701215	192	40720	2132	45	3
436154	436154	ERS044154	ERR065957	CNPY02000001	CNPY02000261	6B		6A	1954	1954	1954	16	1237316	261	46510	2083	41	3
R34-3038	R34-3038	ERS043859	ERR065295	CHBE02000001	CHBE02000186	6B		6A	1930	1930	1930	16	712729	186	55199	2057	46	3
BR1064	EKR1O	ERS069935	ERR129044	COAS02000001	COAS02000225	6C		6C	1692	1692	1692	16	534817	225	34151	2003	31	3
CH2006	WG60C	ERS069962	ERR129071	COBJ02000001	COBJ02000123	6C	NT	NT	547	3288	3288	16	623754	123	54052	2001	43	2
ND6012	P0N8K	ERS070085	ERR129194	COFE02000001	COFE02000225	6C		6C	1692	1692	1692	16	471694	225	39894	1995	31	3
ND6133	6JT2I	ERS070121	ERR124230	CNXY02000001	CNXY02000168	6C		6C	547	547	547	16	771433	168	66394	2109	46	3
R34-3086	R34-3086	ERS043905	ERR067991	CNWO02000001	CNWO02000172	6C		6C	547	547	547	16	718583	172	80401	2107	43	3
BR1074	2VBTK	ERS069938	ERR129047	COAV02000001	COAV02000280	7C		7C	3284	3284	3284	16	582701	280	34224	1953	32	3
ND6000	W9G4K	ERS070078	ERR129187	COFA02000001	COFA02000163	7C		7C	3283	1797	1797	16	575295	163	47752	2018	44	3
BR1113	U3EO1	ERS069955	ERR129064	COBG02000001	COBG02000094	7F		7A	1176	1176	1176	16	515607	94	65255	1925	46	3
132571	132571	ERS044075	ERR069769	CNTM02000001	CNTM02000126	9N		9N	405	405	405	16	726669	126	73240	1988	45	3
436915	436915	ERS044155	ERR065958	CNPZ02000001	CNPZ02000168	9N		9N	66	66	66	16	1343098	168	80750	1989	46	3
MD5007	GJ6MQ	ERS070053	ERR129162	COEF02000001	COEF02000152	9N		9N	1797	66	66	16	512782	152	78813	1987	45	3
ND6123	14YE5	ERS070117	ERR124226	CFJW02000001	CFJW02000135	9N		9N	405	405	405	16	1035462	135	61282	1980	42	3
GL3012	34YLE	ERS069993	ERR129102	COCP02000001	COCP02000142	NT		NT	3288	3288	3288	16	535078	142	57331	2011	47	3
R34-3108	R34-3108	ERS043923	ERR068009	CNWW02000001	CNWW02000174	NT		NT	1905	2011	2011	16	635137	174	58280	1990	43	3
For each sample, the results of serological typing before storage (using the Quellung reaction), after revival (using latex agglutination) and after sequencing (inferred from Illumina read mapping) are tabulated. Similarly, the results of genotyping are shown following the original isolation (using capillary sequencing), after re-isolation and whole genome sequencing (inferred from Illumina read mapping) and after *de novo* assembly (using loci extracted from the assembled contigs). These are used as a check on the integrity of sample handling and processing. The properties of the final draft assemblies are also displayed, including the number of contigs and annotated sequence features they contain.																		
